# circFARP1 enables cancer-associated fibroblasts to promote gemcitabine resistance in pancreatic cancer via the LIF/STAT3 axis

**DOI:** 10.1186/s12943-022-01501-3

**Published:** 2022-01-19

**Authors:** Chonghui Hu, Renpeng Xia, Xiang Zhang, Tingting Li, Yuancheng Ye, Guolin Li, Rihua He, Zhihua Li, Qing Lin, Shangyou Zheng, Rufu Chen

**Affiliations:** 1 Department of General Surgery, Guangdong Provincial People’s Hospital, Guangdong Academy of Medical Sciences, Guangzhou, Guangdong 510080 People’s Republic of China; 2 Guangdong cardiovascular Institute, Guangdong Provincial People’s Hospital, Guangdong Academy of Medical Sciences, Guangzhou, Guangdong 510080 People’s Republic of China; 3 The Second School of Clinical Medicine, Southern Medical University, Guangzhou, Guangdong 510515 People’s Republic of China; 4 Department of Neonatal/General Surgery, Hunan Children’s Hospital, Changsha, Hunan 410007 People’s Republic of China; 5 Department of Pancreatobiliary Surgery, Sun Yat-sen Memorial Hospital, Sun Yat-sen University, Guangzhou, Guangdong 510120 People’s Republic of China; 6 Guangdong Provincial Key Laboratory of Malignant Tumor Epigenetics and Gene Regulation, Sun Yat-Sen Memorial Hospital, Sun Yat-Sen University, Guangzhou, 510120 People’s Republic of China; 7 School of medicine, South China University of Technology, Guangzhou, Guangdong Province 510006 People’s Republic of China; 8 Department of Hepatobiliary, Pancreatic and Splenic surgery, The Sixth Affiliated Hospital of Sun Yat-sen University, Guangzhou, Guangdong 510655 People’s Republic of China; 9 Department of Oncology, Sun Yat-sen Memorial Hospital, Sun Yat-sen University, Guangzhou, Guangdong 510120 People’s Republic of China

**Keywords:** PDAC, CAFs, Chemoresistance, circRNAs, LIF

## Abstract

**Background:**

Cancer-associated fibroblasts (CAFs) are critically involved in gemcitabine (GEM) resistance in pancreatic ductal adenocarcinoma (PDAC). However, the underlying mechanism by which CAFs promote chemotherapy resistance remains unexplored. Here, we explored the role of circRNAs in CAF-induced GEM resistance in PDAC.

**Methods:**

circRNA sequencing and quantitative real-time PCR (qRT–PCR) were utilized to screen CAF-specific circRNAs. The effects of CAF circFARP1 expression on GEM resistance in tumor cells were assessed *in vitro* and *in vivo*. RNA-seq, RNA pulldown, RNA immunoprecipitation, and luciferase reporter assays were used to screen the downstream target and underlying mechanism of circFARP1.

**Results:**

circFARP1 (hsa_circ_0002557), a CAF-specific circRNA, was positively correlated with GEM chemoresistance and poor survival in an advanced PDAC cohort. Silencing or overexpressing circFARP1 in CAFs altered the ability of CAFs to induce tumor cell stemness and GEM resistance via leukemia inhibitory factor (LIF). Mechanistically, we found that circFARP1 directly binds with caveolin 1 (CAV1) and blocks the interaction of CAV1 and the E3 ubiquitin-protein ligase zinc and ring finger 1 (ZNRF1) to inhibit CAV1 degradation, which enhances LIF secretion. In addition, circFARP1 upregulated LIF expression by sponging miR-660-3p. Moreover, high circFARP1 levels were positively correlated with elevated serum LIF levels in PDAC and poor patient survival. Decreasing circFARP1 levels and neutralizing LIF significantly suppressed PDAC growth and GEM resistance in patient-derived xenograft models.

**Conclusions:**

The circFARP1/CAV1/miR-660-3p/LIF axis is critical for CAF-induced GEM resistance in PDAC. Hence, circFARP1 may be a potential therapeutic target for PDAC.

**Supplementary Information:**

The online version contains supplementary material available at 10.1186/s12943-022-01501-3.

## Background

Despite constant progress in the application of multiple therapeutic strategies and expanded research efforts, pancreatic ductal adenocarcinoma (PDAC) remains one of the most aggressive and lethal malignancies [1]. The tumor microenvironment (TME) has been increasingly recognized as a key factor driving cancer development, while strategies aimed at deconstructing the desmoplastic stroma have been largely disappointing owing to the intricate network between tumor cells and the TME [2–4]. In the course of chemotherapy or radiotherapy, tumor cells dynamically adapt to stress via self-mutation and phenotypic transformation [5, 6], but the key roles of various components of the TME in the response to chemo- and radiotherapy remain unclear.

Cancer-associated fibroblasts (CAFs) are the predominant cell type within the tumor stroma; they exhibit heterogeneity and plasticity during cancer evolution and can have tumor-promoting, tumor-restraining or homeostatic functions in PDAC [7]. CAFs induce chemoresistance by mediating the remodeling of the extracellular matrix (ECM) and the reprogramming of metabolism and immune function [8]. The pleiotropic actions of CAFs on cancer cells have been recently revealed, suggesting that various CAF populations have heterogeneous histologic, epigenetic, immunologic, and mechanical signatures with context-dependent influences on cancer [9, 10]. Therefore, to precisely target heterogeneous CAFs that contribute to cancer progression, it is necessary to enhance our understanding of the modulation of CAFs in the TME.

Given the low rate of surgical resection and high rate of recurrence, systemic chemotherapy is the primary treatment option for most PDAC patients, while drug resistance eventually emerges in almost all patients. Recently, chemotherapy resistance driven by the TME has attracted substantial attention, and studies on how CAFs confer chemoresistance by interfering with drug delivery, secreting functional cytokines or inducing immune suppression have been increasingly reported [11]. For years, gemcitabine (GEM)-based regimens have been the standard treatment for advanced PDAC, but the response rate is only 29% for first-line therapy with albumin-bound paclitaxel plus GEM [12]. CAF-induced desmoplastic stroma is thought to create a physical barrier to GEM perfusion. However, an increasing number of studies suggest that limited drug delivery is not the only reason for PDAC chemoresistance [13, 14]. Additionally, recent studies revealed that CAFs contribute to GEM resistance via direct contact and paracrine signaling [15, 16] or by releasing deoxycytidine to sequester GEM in tumor cells [17]. Nevertheless, in light of the heterogeneity of CAFs in tumors and the disappointing results from clinical trials of anti-CAF therapy, the specific upstream signal transduction pathway by which CAFs drive GEM resistance in pancreatic cancers needs to be elucidated.

Circular RNAs (circRNAs) are covalently closed structures with exonuclease resistance that are uniquely generated by a noncanonical splicing event called backsplicing [18]. circRNAs have diverse biological functions; they act as microRNA (miRNA) sponges, protein scaffolds or decoys and can mediate self-translation [19]. circRNAs are now recognized to function as either oncogenes or tumor suppressors in different types of tumors [20], including PDAC [21, 22]. However, the role of circRNAs in CAF-induced GEM resistance in PDAC remains largely unknown. Here, we demonstrated that the overexpression of circFARP1 (hsa_circ_0002557) in CAFs contributed to GEM resistance in PDAC by enhancing the expression and secretion of leukemia inhibitory factor (LIF) in CAFs and thereby inducing the STAT3 signaling pathway in pancreatic cells. These findings provide information to facilitate the development of strategies to selectively target CAF subgroups or specific signaling pathways that mediate GEM resistance in PDAC.

## Methods

### Patients and clinical samples

Tumor biopsy samples were collected from 82 patients with advanced PDAC who underwent GEM-based chemotherapy at the Sun Yat-Sen Memorial Hospital and Guangdong Provincial People's Hospital between 2015 and 2021. The obtained tissues were immediately snap-frozen in liquid nitrogen and transferred at −80 °C for further experiments. All samples were histologically confirmed with PDAC. Progression-free survival (PFS) was defined as the time interval beginning the date of chemotherapy to the date of disease progression event occurrence. All patients provided informed consent, and all related procedures were performed with the approval of Ethical Committee of indicated hospitals.

### Isolation and culture of stromal fibroblasts

Cancer-associated fibroblasts (CAFs) and Primary normal fibroblasts (NFs) were isolated from pancreatic ductal carcinoma and adjacent normal tissues. The Human Tumor Dissociation Kit (130-095-929, Miltenyi Biotec, German) was used for the generation of single cells from dissociated tissues. Fibroblast populations were isolated by differential velocity adherent technique. Primary CAFs and NFs were culture in Dulbecco’s modified Eagle’s medium (DMEM, GBICO) plus 15% fetal bovine serum (FBS, GBICO) and 1% penicillin-streptomycin at 37°C in humidified air with 5% CO_2_.

### Conditioned medium preparation and co-culture

About 2 × 10^6^ stable transfected CAFs were cultured in a 10 cm cell culture dish for 48 h. The culture medium was collected and centrifuged to removed cell pellets. Panc-1 and MiaPaCa-2 were culture with the conditioned medium (CM) for 2 weeks and then were subjected for cytological experiments.

### Cell lines

PDAC cell lines Panc-1 and MiaPaCa-2 were obtained from American Type Culture Collection (ATCC) and cultured at 37°C condition with 5% CO_2_ and at least 95% humidity atmosphere with Dulbecco’s Modified Eagle Medium (DMEM, Gibco). Medium was added 10% fetal bovine serum (FBS, Gibco).

### RNA isolation and quantitative real-time PCR (qRT-PCR)

Total RNA from frozen tissues and cultured cell lines was extracted with TRIzol reagent (Life, USA) and weas reverse transcribed into cDNA using PrimeScript RT Reagent Kit (Takara, Japan). The qRT-PCR analysis was performed with TB Green Premix Ex TaqTM kit (Takara, Japan) on Light Cycler 480 Detection System (Roche, Switzerland), The 2 −ΔΔCt method was used to analyze the relative levels of target gene. All primer sequences for the qRT-PCR assay were listed in supplementary Table S1.

### Cell transfection

The full length of circFARP1 sequence was cloned into pCD-ciR vector by IGE (Guangzhou, China). shRNA targeting circFARP1, LIF or CAV1 were ordered from IGE. The miR-660-3p mimic and inhibitor were purchased from IGE. The sequences of shRNA are provided in supplementary Table S2.

### RNase R digestion and actinomycin D assay

For RNase R digestion assay, total RNA of NFs and CAFs were treated with or without 5 U/μg RNase R (RNR07250, Epicenter Technologies) and incubated at 37℃ for 30 min. For actinomycin D assay, cells were treated with 2μg/mL actinomycin D (Sigma, USA) for 0 h, 4 h, 8 h, 12h and 24 h. And qRT-PCR was used to detected circFARP1 and FARP1 expression levels. The experiments were performed three times.

### CCK-8 assay

For drug response of PDAC cells, 4000 of treated pancreatic cells were seeded in 96-well plates per well. The next day, fresh medium containing gemcitabine at a gradient concentration of 0, 0.001, 0.01, 0.1, 1, 10, 100, 1000μM was added into cells and incubated for 72 h. The cells were incubated with 10μl CCK-8 solution (Dojindo, Japan) at 37°C for 2h. Then, the absorbance was measured using a microplate reader (Tecan Trading AG, Switzerland) at 450 nm. The degree of drug response for tumor cells was determined by the half-maximal inhibitive concentration (IC50), which was calculated with software GraphPad Prism 8.0

For cell proliferation, 4000 pretreated cells were seeded in 96-well plates per well and incubated for 4 days. The cell viability was measured daily by reading the absorbance at 450 nm.

### EdU labelling assay

5-ethynyl-2’-deoxyuridine (EdU) immunofluorescence assay was performed with BeyoClick™ EdU-555 kit (Beyotime, Guangzhou) according to the manufacturer’s instructions. Briefly, Indicated CAFs were seeded in 96-well plate and incubated with EdU for 3 h. After fixation and permeabilization, cells were stained with anti-EdU reagents and DAPI. Images were acquired by fluorescence microscopy.

### Scratch wound healing assay

2×10^5^ indicated CAFs were cultured in 24-well plate to reach confluence. The cells were scratched with a sterile 10 μL pipette tip and incubated with FBS-free culture medium. The degree of cell migration was monitored at 0 and 24 h post-scratching.

### Collagen contraction assay

1×10^5^ indicated CAFs were mixed with 200 μl of collagen containing 168.75 μL culture medium, 0.72 μL NaOH, and 31.25 μL Rat Tail Collagen I (Corning) and seeded in 24-well plates. Culture medium was added on top of the gels after polymerization. Plates were scanned 24 h after plating and percentage of contraction was calculated using the formula: Area (well)-Area (gel)/Area (well).

### Colony formation assay

500 Panc-1 or MiaPaCa-2 cells with indicated treatments were seeded into 6-well plates and allowed to attach for 24h. After treated with gemcitabine (5μM) for 2 days, the media was replaced with complete media and the cells were cultured for 2 weeks. Then the colonies were fixed in 4% paraformaldehyde for 20 min, followed by staining with 0.1% crystal violet. Colonies were then manually counted. Three different independent experiments were performed.

### Sphere formation assay

PANC-1 and MiaPaCa-2 cells were plated in 96-well ultra-low attachment plates (Corning, NY, USA) at a density of 1000 cells per well. Cells were maintained in serum-free DMEM/F-12 supplemented with 20ng/ml human recombinant epidermal growth factor, 20ng/ml basic fibroblast growth factor, and 1× B27 serum substitute; all from Invitrogen, Carlsbad, CA, USA. After incubated at 37°C in 5% CO2 for 2 weeks, spheres with larger than 50μm in diameter were counted.

### Annexin V-FITC assay

The Annexin V-FITC Apoptosis Detection Kit was applied to detected cell apoptosis in line with the manufacturer’s instructions. In brief, cells were digested with trypsin and washed by PBS twice. Then cells were dual-stained with PI and Annexin V-FITC, using the Annexin V/FITC kit (Thermo Fisher Scientific, Shanghai, China). The analysis was carried out in the BDTM LSRII flow cytometer (BD Biosciences). and the data were measured with the Cell Quest (BD Bioscience, San Jose, CA, United States) software.

### Enzyme-linked immunosorbent assay (ELISA)

Concentration of cytokines were determined by using human LIF ELISA Kit (BMS242, Thermo, USA) according to the manufacturer's instructions. In brief, stable transfected CAFs were cultured with serum-free media when reaching 80% of confluency. After 24 h, 100 µl of indicated CAFs medium was collected and incubated with plates at 37℃ for 90 min. Then detection antibody, streptavidin-HRP and TMB were added in order. The absorbance of each well was measured at 450 nm with SPARK 10 M spectrophotometer (Tecan, Austria).

### Western Blotting

Protein was extracted from the cells using RIPA lysis buffer (CWBIO, China), followed by subjected to SDS-polyacrylamide gels and transferred to polyvinylidene difluoride membranes. The membranes were blocked with 5% BSA for 1 hour at room temperature. Corresponding primary antibodies including anti-LIF (1:500, ab138002, Abcam), anti-STAT3 (1:1000, 9139, CST), anti-p-STAS3(Tyr705) (1:1000, 9145, CST), anti-CAV1 (1:1000, ab32577, Abcam), anti-SOX2 (1:1000, ab92494, Abcam), anti-ABCC2 (1:1000, ab172630, Abcam), anti-CDA (1:1000, ab222515, Abcam), anti-ZNRF1 (1:1000, ab175125, Abcam), anti-ubiquitin (1:1000, 3936, CST), anti-GAPDH (1:1,000, abs132004, Absin) were added to the membrane at 4℃ overnight. HRP-conjugated secondary antibodies were used. The protein bands were visualized by ECL detection system (Millipore, Germany).

### Fluorescence in situ hybridization (FISH)

FISH was performed using a In Situ Hybridization Kit (Gene Pharma, Guangzhou, China) according to the manufacturer's instructions. Cy3-labeled circFARP1 and Cy5-labeled hsa-miR-660-3p probes (Gene Pharma, Guangzhou, China) were hybridized with cells overnight at 37℃. All images were captured by confocal microscopy. The targeted sequences of probes are provided in supplementary Table S2.

### RNA in situ hybridization (ISH)

circFARP1 expression in PDAC tissues was detected by ISH analysis using ISH Detection kit (MK1032, BOSTER, China) as manufacture’s instruction. In brief, after deparaffinization, rehydration, and digestion, specimens were incubated with digoxin-labeled circFARP1 probes for 18 h at 40°C. followed with the incubation of anti-digoxin antibody at 37℃ for 2h, BCIP/NBT was used to for the colorimetric detection of circFARP1. The circFARP1 probe sequence was list in Supplementary Table S2.

The staining scores of circFARP1 were determined based on both staining intensity and number of positive cells. Scoring for staining intensity was as follow: 0 (none), 1 (light blue), 2 (bule) and 3 (dark bule). Scoring for ratio of circFARP1 positive cells was as follow: 1 (<25%), 2 (25-50%), 3 (50-75%), 4 (75-100%). The final score was equal to multiply staining intensity and proportion of positively stained cells. The expression of circFARP1 was evaluated by final score, with a cut-off point of <4 versus ≥4.

### Immunohistochemistry (IHC)

Histologic sections from formalin-fixed, paraffin-embedded tissues were subjected to antigen retrieval in citrate buffer for 15min, followed by blocking in normal goat serum for 30min. Then tissue sections were incubated with primary antibody as follow: anti-α-SMA (1:200, 67735-1-Ig, proteintech),anti-Ki-67(1:200, ab15580, abcam), anti-CAV1(1:200, ab32577, Abcam), anti-LIF(1:200, ab138002, Abcam) at 4°C overnight. Avidin–biotin peroxidase detection systems with DAB substrate were used to mark the locations of antigens, followed by counterstaining with hematoxylin. Immunohistochemical signal intensity and positively stained field of tissue sections were evaluated and scored independently by two observers.

### RNA pull-down assay

The biotinylated probes targeting junction sites of circFARP1 were synthesized by IGE (Guangzhou, China). 1×10^7^ CAF cells were harvested, wash with ice-cold PSB twice, and lysed with co-IP buffer. The supernatant was extracted after centrifugation and incubated with 3 μg biotinylated probes at 4℃ overnight. Streptavidin-coated magnetic beads (Invitrogen, Waltham, MA, USA) were added to the mixture and incubated at room temperature for 1 h. The captured protein or RNA were eluted from the magnetic beads and analyzed by mass spectrometry, western blotting, or qRT-PCR. For mass spectrometry, the bound proteins were subjected SDS-PAGE gel and visualized by Silver Staining Kit (24612, Thermo, USA) as manufacture’s instruction. The different band was cut and analyzed by mass spectrometry. The sequences of probes are shown in in supplementary Table S2.

### Luciferase reporter assay

The circFARP1/LIF wild-type or mutant plasmids and miR-660-3p mimic were co-transfected into CAFs cells using Lipofectamine 3000 (Invitrogen, USA) according to the manufacturer’s protocol. Then the transfected Cells were seeded into 96-well plates and luciferase activities were determined by dual-luciferase reporter assay system (Promega, USA) according to the manufacturer's instructions.

### Xenograft tumor model experiments

For mouse subcutaneous xenograft model, 5 × 10^6^ Panc-1 cells alone or mixed with stable transfected CAFs at a ratio of 1:1, were injected into the right flank of 4-weeks-old the BALB/c nude mice. The animals of each group (*n* =5) were assigned to gemcitabine (50 mg/kg, once every four days, intraperitoneally) for 24 days when the tumors reached approximately 3mm in diameter, and tumor size was monitored meantime. All the mice were sacrificed five weeks later. Both maximum (L) and minimum (W) lengths of the tumor were measured using a slide caliper, and the tumor volume was calculated as (LW^2^)/2.

For mouse PDX models, primary tumor specimens collected from GEM-resistant PDAC patients who underwent surgery were propagated as subcutaneous tumors in 4-week-old NSG mice (F1). Xenografts from F1 mice were cut into small pieces and then implanted into other mice (F2). When tumors grew up to about 1500 mm3, they were excised and cut again into small pieces and transplanted to other mice (F3). The combined treatment of In vivo siRNA/ neutralizing antibodies against LIF and GEM chemotherapy was performed when xenografts volume reached about 200 mm3. In vivo-optimized si-Ctrl (5 nmol, intra-tumor injection), si-circFARP1 (5 nmol, intra-tumor injection) or LIF antibody (10 mg/kg, intra-venous injection). Tumor volume was monitored every week and tumor were further analyzed by IHC and qRT-PCR. The chemotherapy responses were assessed refer to the human clinical evaluation standard. Complete Response (CR) was defined as disappearance of tumor; Partial Response (PR) was defined as at least a 30% reduction of tumor volume; Progressive Disease (PD) was defined as at least a 20% of tumor volume; Stable Disease (SD) was defined as no sufficient to qualify as PR and PD. CR and PR were classified as GEM-sensitive, while SD and PD were classified as GEM-resistant.

Animal experiments were conducted according to guidelines approved by the Animal Experimental Research Ethics Committee of South China University of Technology.

### RNA immunoprecipitation (RIP)

RIP was performed by Magna RIP™ RNA-Binding Protein Immunoprecipitation Kit (Millipore) according to the manufacturer’s protocol. Briefly, cells were washed twice with ice-cold PBS, followed by cell lysis using RIP lysis buffer. Magnetic beads were washed twice with RIP wash buffer, followed by incubation with 2 μg antibody against CAV1 or rabbit anti-IgG as a negative control for 30 mins at room temperature. Immunoprecipitation was performed by incubating cell lysate with the magnetic bead-antibody complex overnight at 4°C. Then the beads were washed six times with RIP wash buffer. The precipitated RNAs were eluted and further analyzed by qRT-PCR assays.

### Immunoprecipitation

Cells were lysed in IP lysis buffer and protease inhibitors. For immunoprecipitation, indicated antibody or rabbit anti-IgG as the control was added to the lysates and incubated overnight at 4 °C. Then Dynabeads Protein A /G (10002D/10003D, Invitrogen) was added and then incubated for 3h at 4 °C. The precipitated proteins were analyzed by western blotting.

### RNA-seq

Total RNA was isolated and purified using TRIzol (Life, USA) following the manufacturer's procedure. After the quality inspection of Agilent 2100 Bioanalyzer (Agilent, USA) and NanoPhotometer (Implen, Germany), ribosomal RNA was removed from 1 μg total RNA. VAHTS Universal V6 RNA-seq Library Prep Kit for Illumina (Vazyme, China) was used for lncRNA library construction following the manufacturer's protocol. Each library was sequenced on an Illumina Novaseq 6000 (Illumina Corporation, USA) in 150PE mode following the vendor's recommended protocol by Guangzhou Huayin Health Medical Group CO.,Ltd. (Guangzhou, China).

### Statistical Analysis

All experimental data were expressed as mean ± standard deviation (SD) using GraphPad Prism 8.0. The differences between parametric variables were determined by Student’s t-test or one-way analysis of variance (ANOVA), and nonparametric variables were determined by Mann-Whitney U test. Statistical significance of survival was estimated by Kaplan-Meier analysis and the log-rank test, and correlation analysis was performed by two-sided Pearson’s correlation. Correlation analysis was examined with two-sided Pearson’s correlation. *p* <0.05 was used as an indicator of statistical significance.

## Results

### Identification of a CAF-specific circRNA, circFARP1, that correlated with GEM resistance in PDAC

**Fig. 1:**
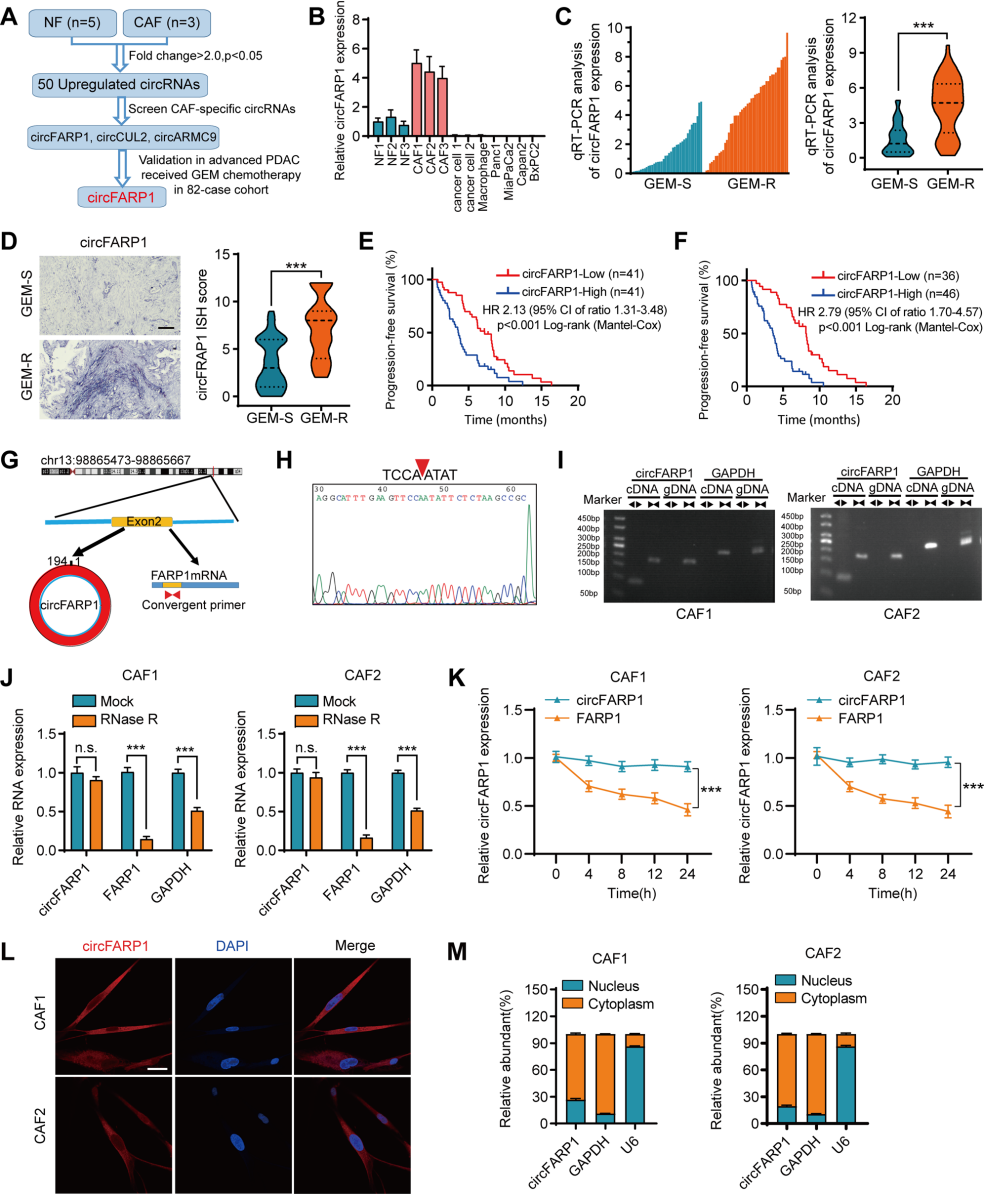
circFARP1 is overexpressed in CAFs and is associated with GEM chemoresistance and poor survival in advanced PDAC. **A** Schematic illustration of the identification of circFARP1 upregulated in CAFs. **B** qRT–PCR analysis of circFARP1 expression in NFs, CAFs, primary cancer cell, macrophages, and PDAC cell lines. **C** Quantification of circFARP1 expression by using qRT–PCR in GEM-S (*n* = 38) and GEM-R (*n* = 44) PDAC tissues. The left panel shows the plot of circFARP1 expression in each tissue. Right panel shows the expression as violin plots. **D** Representative images (left) and quantification (right) of circFARP1 by using ISH in GEM-S (*n* = 38) and GEM-R (*n* = 44) PDAC tissues. Scale bars, 50 μm. **E-F** Kaplan–Meier survival curves for advanced PDAC patients who received GEM-based chemotherapy with high or low circFARP1 expression evaluated by qRT–PCR (**E**) or ISH (**F**). A univariate Cox regression model was used to calculate the hazard ratio (HR). **G** Schematic illustration showing the genomic loci of circFARP1. CircFARP1 was generated by exons 2 of FARP1. **H** The back-splice junction of circFARP1 was identified by Sanger sequencing. **I** cDNA and gDNA of CAF1 and CAF2 were amplified with convergent and divergent primers. GAPDH was as the negative control. **J** PCR analysis of circFARP1, FARP1, and GAPDH expression in CAF1 and CAF2 cells treated with or without RNase R. n.s., not significant. **K** qRT–PCR analysis of circFARP1 and FARP1 mRNA in CAF1 and CAF2 cells treated with actinomycin D at the indicated time points. **L** Representative FISH images for circFARP1 in CAF1 and CAF2. Scale bars, 100 μm. **M** Subcellular fractionation assays of circFARP1 in CAF1 and CAF2. Data are expressed as the mean ± SD. ***p<0.001

To characterize upregulated circRNAs in CAFs that mediate GEM resistance in PDAC, we first analyzed RNA-seq in CAFs and paired normal fibroblasts (NFs) (GSE172096) and identified 50 upregulated circRNAs in CAFs (Fig. 1A). Next, quantitative real-time PCR (qRT–PCR) was performed to screen the expression of CAF-specific circRNAs, and the results showed that 3 circRNAs, circFARP1, circCUL2 and circARMC9, were significantly expressed in CAFs but not in cancer cells or other stromal cells (Fig. 1B and Fig. S1A-B). Finally, validation in a cohort of 82 advanced PDAC patients who received chemotherapy containing GEM showed that only circFARP1 was significantly overexpressed in patients who did not respond to GEM treatment (indicated as GEM-R) compared with those sensitive to GEM treatment (indicated as GEM-S) (*p* <0.001, by using qRT–PCR) (Fig. 1C-1D and Fig. S1C-D). Patients with higher circFARP1 expression had poorer progression-free survival (PFS) than those with lower circFARP1 expression (*p* <0.001, by using ISH, *p* <0.001, by using qRT–PCR) (Fig. 1E-1F).

circFARP1 is formed by the circularization of exon 2 of the FARP1 gene, which has a length of 194 nt according to circBase (Fig. 1G). Accordingly, Sanger sequencing confirmed the head-to-tail splicing site of circFARP1 (Fig. 1H). Nucleic acid electrophoresis showed that circFARP1 could be amplified by outward-facing divergent primers from cDNA but not from gDNA in CAFs (Fig. 1I). RNase R and actinomycin D treatment showed that circFARP1 had potential resistance to RNase R and was more stable than its linear form (Fig. 1J-K). Fluorescence *in situ* hybridization (FISH) and subcellular fractionation assays revealed that circFARP1 was predominantly located in the cytoplasm (Fig. 1L-M).

### circFARP1 is critical for CAFs to induce GEM resistance in PDAC cells

To investigate the role of circFARP1 in CAF-induced GEM resistance, we overexpressed circFARP1 in CAFs with a circFARP1 plasmid or silenced circFARP1 expression with two shRNAs (sh-circFARP1#1 and sh-circFARP1#2) without altering FARP1 expression (Fig. S2A-B). Next, we explored the effects of circFARP1 on the intrinsic characteristics of CAFs. We observed no significant changes in the morphology and proliferation of CAFs transfected with circFARP1 or lenti-circFARP1-shRNA (Fig. S3A-F). However, circFARP1-overexpressing CAFs displayed higher migration skills than the control, while circFARP1 knockdown had the opposite effect (Fig. S3G-J). Interestingly, we found that upregulation of circFARP1 promoted the collagen gel contraction, while circFARP1 depletion attenuated this effect (Fig. S3K-L).

**Fig. 2:**
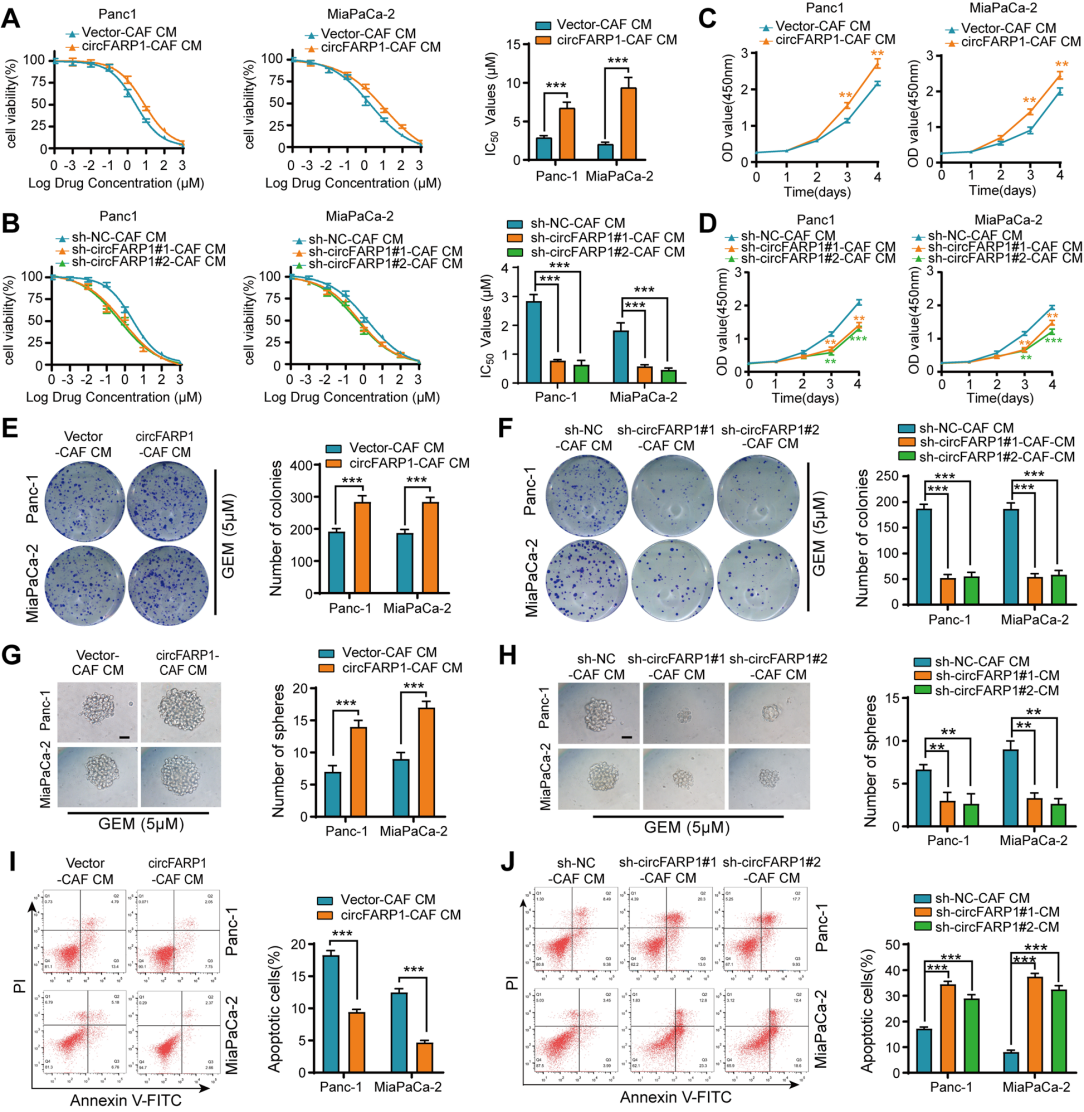
circFARP1 is critical for CAFs to induce GEM resistance in PDAC cells. Panc-1 and MiaPaCa-2 cells were grown in conditioned medium (CM) from CAFs stably transfected with empty vector, circFARP1, lenti-NC-shRNA, or lenti-circFARP1-shRNA for 2 weeks and then subjected to the indicated experiments. **A-B** GEM IC50 of Panc-1 and MiaPaCa-2 determined by constructing a dose–response curve. **C-D** Cell proliferation of Panc-1 and MiaPaCa-2 cells was detected by CCK-8 assay with the indicated treatment. **E-F** Colony formation assays of Panc-1 and MiaPaCa-2 cells under GEM treatment (5 μM). **G-H** Spheroid growth assay of Panc-1 and MiaPaCa-2 cells under GEM treatment (5 μM). Scale bars, 50 μm. **I-J** Flow cytometry analysis of GEM-induced (10 μM) apoptosis in Panc-1 and MiaPaCa-2 cells. Data are expressed as the mean ± SD. **p<0.01 and ***p<0.001

Then, the indicated CAF-derived conditioned medium (CAF-CM) was collected and incubated with the PDAC cell lines. The Cell Counting Kit-8 (CCK-8) cytotoxicity assay indicated that upregulating circFARP1 in CAFs increased the half-maximal inhibitory concentration (IC_50_) of GEM in Panc-1 and MiaPaCa-2 cells following treatment with CAF-CM (Fig. 2A). Conversely, silencing circFARP1 abrogated CAF-induced GEM resistance in tumor cells (Fig. 2B). CM from circFARP1-overexpressing CAFs markedly increased the proliferation rate of tumor cells (Fig. 2C). Colony formation assays showed that tumor cells incubated with medium from circFARP1-overexpressing CAFs had a higher survival rate than control CAFs after exposure to GEM (Fig. 2E). Cancer stem cells play a pivotal role in chemoresistance. In agreement, sphere formation assays demonstrated that medium from circFARP1-overexpressing CAFs enhanced the self-renewal ability of tumor cells (Fig. 2G). Additionally, flow cytometry assays demonstrated that upregulating circFARP1 enhanced the ability of CAFs to induce apoptosis resistance in tumor cells under GEM treatment conditions (Fig. 2I). In contrast, knockdown of circFARP1 in CAFs dramatically attenuated these effects (Fig. 2D, F, H and J). Collectively, these data suggest that circFARP1 plays a critical role in CAF-induced resistance to GEM.

### circFARP1 enhances the expression and secretion of LIF in CAFs to induce chemoresistance

**Fig. 3:**
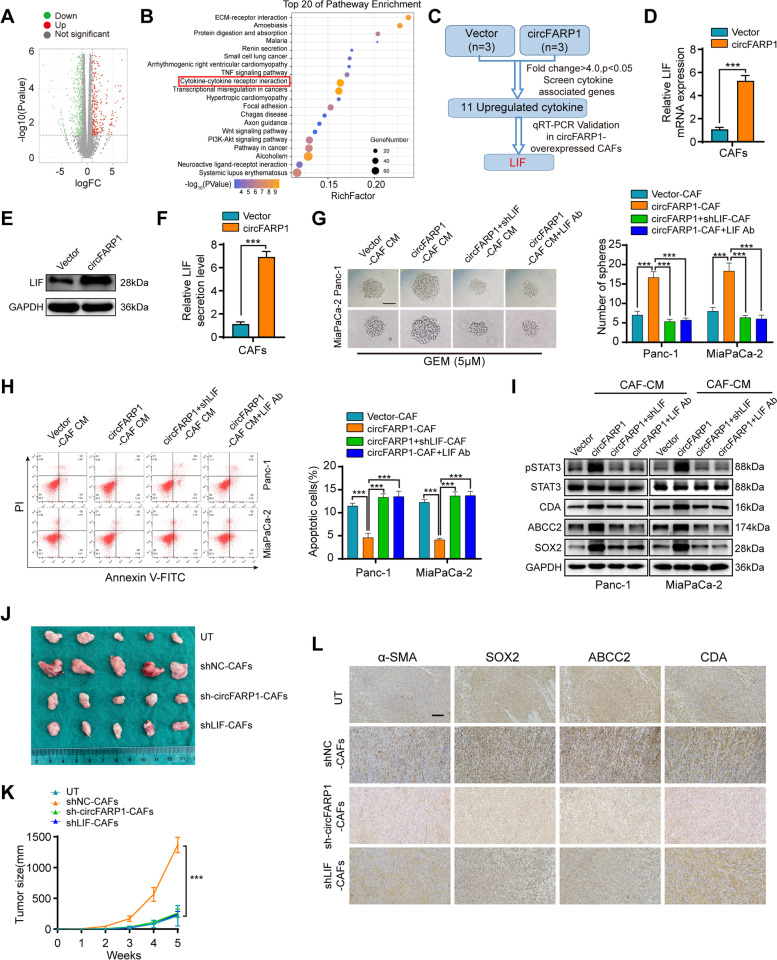
circFARP1 enhances the expression and secretion of LIF in CAFs. **A** Plot showing the sums of the expression levels of genes regulated by circFARP1. **B** Top 20 enriched pathways of differential mRNA expression between CAFs and circFRAP1-overexpressing CAFs. **C** Flow chart for the identification of LIF as the downstream target of circFARP1. **D-F** The mRNA level (**D**), protein level (**E**), and secretion level (**F**) of LIF in CAFs transfected with vector or circFARP1. **G-I** Panc-1 and MiaPaCa-2 cells were grown in conditioned medium (CM) from CAFs transfected with empty vector or circFARP1 for 2 weeks and subjected to the indicated experiments. Lenti-LIF shRNA or a neutralizing antibody against LIF was used to deplete LIF in CAF-CM. (**G**) Sphere formation assays of Panc-1 and MiaPaCa-2 cells with the indicated treatments. Scale bars, 50 μm. (**H**) Flow cytometry analysis of GEM-induced (10 μM) apoptosis in Panc-1 and MiaPaCa-2 cells with the indicated treatment. (**I**) western blot analysis of pstat3/stat3, ABCC2, CDA, and SOX2 protein expression in Panc-1 and MiaPaCa-2 cells with the indicated treatment. **J-L** Panc-1 cells were subcutaneously coinjected with or without CAFs stably transfected with lenti-NC-shRNA, lenti-circFARP1-shRNA or lenti-LIF-shRNA into nude mice followed by GEM treatment (50 mg/kg). UT, untreated Panc-1. **J** Representative images of xenograft tumors of each group. **K** Tumor growth curve were shown. **L** Representative images of IHC for α-SMA, SOX2, ABCC2, and CDA in xenograft tumors. Scale bars, 50 μm. Data are expressed as the mean ± SD. ***p<0.001

To clarify how circFARP1 exerts its function in CAFs, we performed next-generation sequencing to compare gene expression profiles following circFARP1 upregulation in CAFs (Fig. 3A). Kyoto Encyclopedia of Genes and Genomes (KEGG) assays revealed an enrichment of the cytokine pathway in circFARP1-overexpressing CAFs; therefore, we focused on cytokine alterations (Fig. 3B). As shown in Fig. 3C, a total of 11 cytokine genes were differentially expressed by at least 4-fold in circFARP1-overexpressing CAFs versus the control. Further validation by qPCR showed that LIF was the most significantly elevated gene in CAFs (Fig. 3D, Fig. S4A). Western blotting and ELISA further confirmed that upregulated circFARP1 in CAFs increased the expression and secretion level of LIF (Fig. 3E-F). Depletion of circFARP1 in CAFs greatly decreased the expression and secretion of LIF (Fig. S4B-C). Consistently, silencing or neutralizing LIF in circFRAP1-overexpressing CAFs reduced their effect to enhance the sphere formation and apoptosis resistance of tumor cells under GEM treatment (Fig. 3G-H). Western blotting assays indicated that CAF-derived LIF may activate STAT3 phosphorylation to induce GEM resistance (Fig. 3I). The mechanisms of STAT3 pathway-mediated GEM resistance have been reported to include drug efflux (e.g., ABCC2), detoxification (e.g., CDA), stemness (e.g., SOX2) and antiapoptotic proteins (e.g., CDA). Western blot assays demonstrated that the protein levels of ABCC2, CDA, and SOX2 were increased by CAF-derived LIF (Fig. 3I). Moreover, both LIF depletion and LIF neutralizing antibody abolished the effect of circFARP1-overexpressing CAFs (Fig. 3G-I).

Next, we further confirmed the role of the circFARP1/LIF axis in GEM resistance in pancreatic xenograft tumor models. Panc-1 cells were coinjected with or without CAFs into the buttocks of nude mice. When the tumor diameter reached 3 mm, the mice received five intraperitoneal injections of 50 mg/kg GEM at 4-day intervals. Coinjection with CAFs resulted in a reduced response to chemotherapy, with larger tumor sizes and weights and an increased proliferation rate in tumor cells evaluated by Ki-67 assay (Fig. 3J-K, Fig. S4D-F). IHC analysis confirmed that CAFs significantly enhanced SOX2, CDA, and ABCC2 expression (Fig. 3L). Moreover, this effect was reversed by transfecting either lenti-shcircFARP1 or lenti-shLIF into CAFs (Fig. 3J-L, Fig. S4D-F). Our data suggest that circFARP1 potentiates the capacity of CAFs by enhancing the expression and secretion of LIF.

### circFARP1 directly interacts with CAV1 and inhibits its degradation

**Fig. 4:**
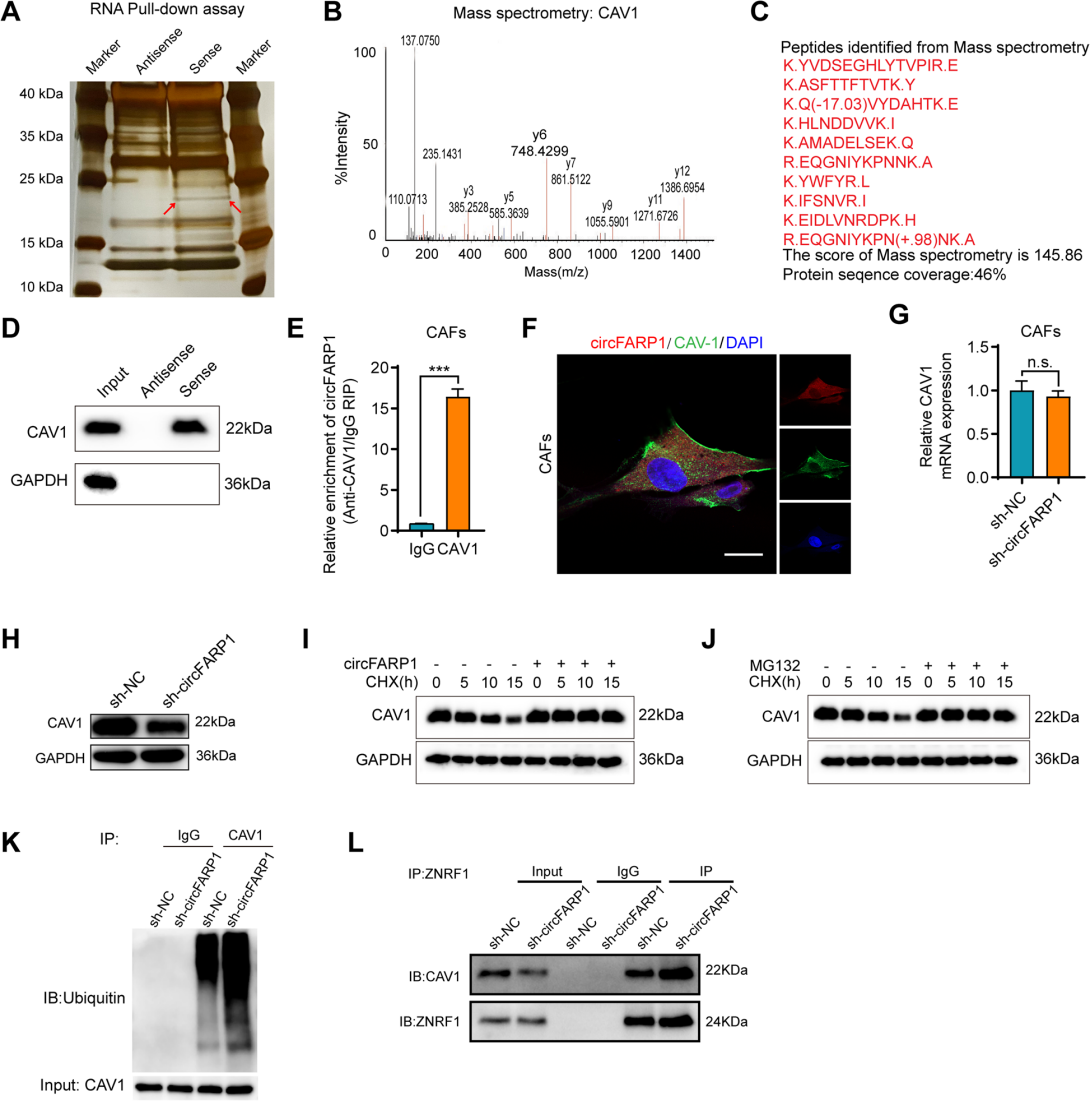
circFARP1 directly interacts with CAV1 and inhibits its degradation. **A** Silver staining for RNA pull-down assay with the specific biotin-labeled circFARP1 probe in CAF lysates. Red arrows indicate the unique differential band precipitated by the circFARP1 probe. **B-D** Mass spectrometry (**B**-**C**) and western blot (**D**) analysis of proteins in unique differential bands. CAV1 was identified as a candidate protein interacting with circFARP1. **E** RNA immunoprecipitation (RIP) assays in CAFs using IgG and CAV1 antibodies. The relative enrichment of circFARP1 was calculated by qRT–PCR. **F** Dual RNA-FISH and immunofluorescence staining assay indicating the colocalization of circFARP1 (red) and CAV1 (green), with nuclear staining with DAPI (blue). Scale bars, 100μm. **G-H** The mRNA and protein levels of CAV1 in CAFs transfected with sh-NC or sh-circFARP1. n.s., no significant. **I** Western blot of the expression kinetics of CAV1 in CAFs transfected with empty vector or circFARP1 plasmid and treated with CHX (100 μg/ml) for 0 h, 5 h, 10 h, or 15 h (GAPDH as a control). **J** western blot of the expression kinetics of CAV1 in CAFs treated with or without MG132 (20 μM) after CHX (100 μg/ml) treatment for 0 h, 5 h, 10 h, and 15 h (GAPDH as a control). **K** Immunoprecipitation of CAV1 protein in lysates from CAFs with or without circFARP1 silencing, followed by immunoblotting with an anti-ubiquitin antibody. MG132 (20 μM) was added before cell lysis to inhibit CAV1 degradation. **L** Immunoprecipitation assays demonstrating the interaction of ZNRF1 and CAV1 in CAFs with the indicated treatments. Data are expressed as the mean ± SD. ***p<0.001

To understand how circFARP1 mediates the expression and secretion of LIF, we performed an RNA pulldown assay to determine the potential binding proteins in CAFs using biotinylated circFARP1 probes targeting the back-spliced sequence. Following silver staining, mass spectrometry analysis and western blotting assay, we discovered that CAV1 was enriched on circFARP1 (Fig. 4A-D). The interaction between circFARP1 and CAV1 was further confirmed by an RNA immunoprecipitation (RIP) assay (Fig. 4E). FISH-immunofluorescence (IF) assays revealed the colocalization of circFARP1 and CAV1 in the cytoplasm (Fig. 4F). circFARP1 depletion in CAFs did not alter CAV1 mRNA expression levels but decreased the protein levels of CAV1 (Fig. 4G-H). The cyclohexamide (CHX) assay suggested that the protein level alteration was attributed to the degradation of CAV1 protein, which was suppressed under MG132 treatment (Fig. 4I-J). Consistently, a marked increase in ubiquitinated CAV1 was detected in circFARP1-depleted cells compared with negative control cells (Fig. 4K). It has been reported that the E3 ubiquitin-protein ligase zinc and ring finger 1 (ZNRF1) can promote CAV1 degradation [23]. Coimmunoprecipitation (Co-IP) assays revealed that circFARP1 blocked the interaction between CAV1 and ZNRF1 (Fig. 4L). These results indicate that circFARP1 directly binds to CAV1 and suppresses the ubiquitination of CAV1.

### circFARP1 enhances LIF secretion via CAV1

**Fig. 5:**
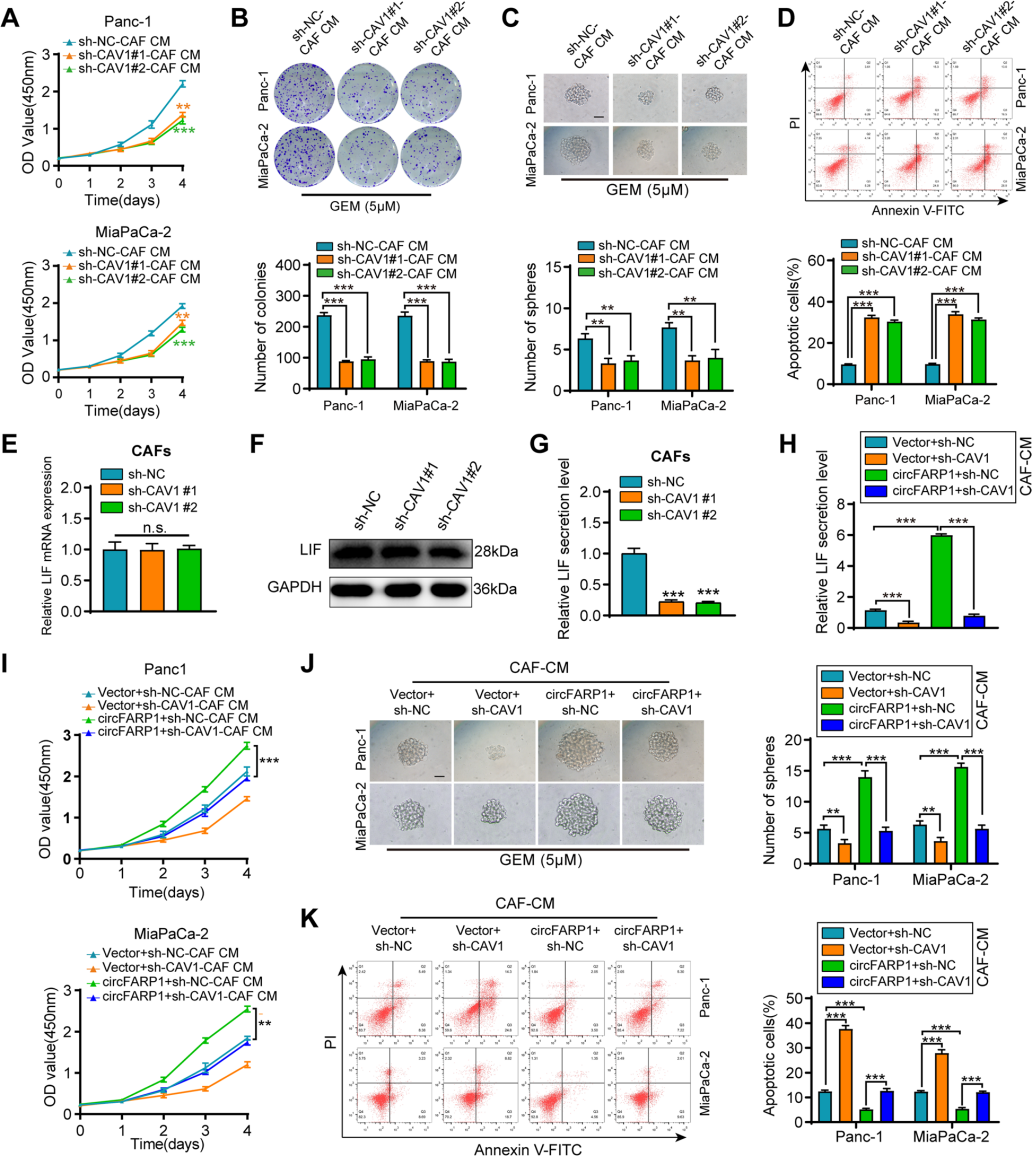
circFARP1 enhances LIF secretion via CAV1 in CAFs. **A-D** Panc-1 and MiaPa-2 cells were grown in CM from CAFs transfected with lenti-NC-shRNA or lenti-CAV1-shRNA for 2 weeks and then subjected to subsequent experiments. **A** Cell viability of pretreated Panc-1 and MiaPaCa-2 cells was detected by CCK-8 assay. **B** Colony formation assays of Panc-1 and MiaPaCa-2 cells under GEM treatment (5 μM). **C** Sphere forming assays of Panc-1 and MiaPaCa-2 cells under GEM treatment (5 μM). Scale bars, 50μm. **D** Flow cytometry analysis of GEM-induced (10 μM) apoptosis in Panc-1 and MiaPaCa-2 cells. **E-G** The mRNA expression, protein level, and secretion level of LIF in CAFs with CAV1 silencing or not. n.s., no significant. **H** ELISA of the supernatant LIF level in CAFs transfected with lenti-circFARP1 and lenti-CAV1-shRNA alone or together. **I-K** Panc-1 and MiaPa-2 cells were grown in CM from CAFs transfected with lenti-circFARP1 and lenti-CAV1-shRNA alone or together for 2 weeks and then subjected to subsequent experiments. **I** The viability of Panc-1 and MiaPaCa-2 cells was detected by CCK-8 assay. **J** Representative images and quantification of sphere formation ability of Panc-1 and MiaPaCa-2 cells under GEM treatment (5 μM). Scale bars,50μm. **K** Flow cytometry analysis of GEM-induced (10 μM) apoptosis in Panc-1 and MiaPaCa-2 cells. Data are expressed as the mean ± SD. **p<0.01, ***p<0.001

As expected, silencing CAV1 in CAFs attenuated their ability to promote pancreatic cell proliferation, colony formation, sphere formation, and apoptosis resistance, similar to the cancer phenotypes induced by circFARP1 or LIF depletion (Fig. 5A-D). Consistently, the protein levels of pSTAT3, SOX2, CDA, and ABCC2 in cancer cells were decreased after silencing CAV1 in CAFs (Fig. S5A). Given that CAV1 is the main integral protein of caveolae and is essential for caveolae-related endocytosis and exocytosis transport processes [24–26], we performed qRT–PCR, western blotting and ELISA to detect LIF expression in CAV1-depleted CAFs. The results showed no significant difference in the mRNA and protein levels of LIF regardless of CAV1 depletion, while the secretion of LIF was greatly decreased after CAV1 depletion (Fig. 5E-G). Then, we cotransfected CAFs with circFARP1 and anti-CAV1 shRNA. The increases in LIF secretion by circFARP1 overexpression were blocked by CAV1 depletion (Fig. 5H). Consistently, CAV1 depletion greatly reversed the promoting effect of circFARP1 overexpression on tumor cell proliferation, sphere formation, and apoptosis resistance in CAFs (Fig. 5I-K). The increases in the protein levels of pSTAT3, SOX2, CDA, and ABCC2 induced by circFARP1-overexpressing CAF-derived CM were blocked by CAV1 depletion (Fig. S5B). These data indicated that circFARP1 enhances LIF secretion to induce GEM resistance via CAV1.

### circFARP1 enhances LIF expression by functioning as a miR-660-3p sponge in CAFs

**Fig. 6:**
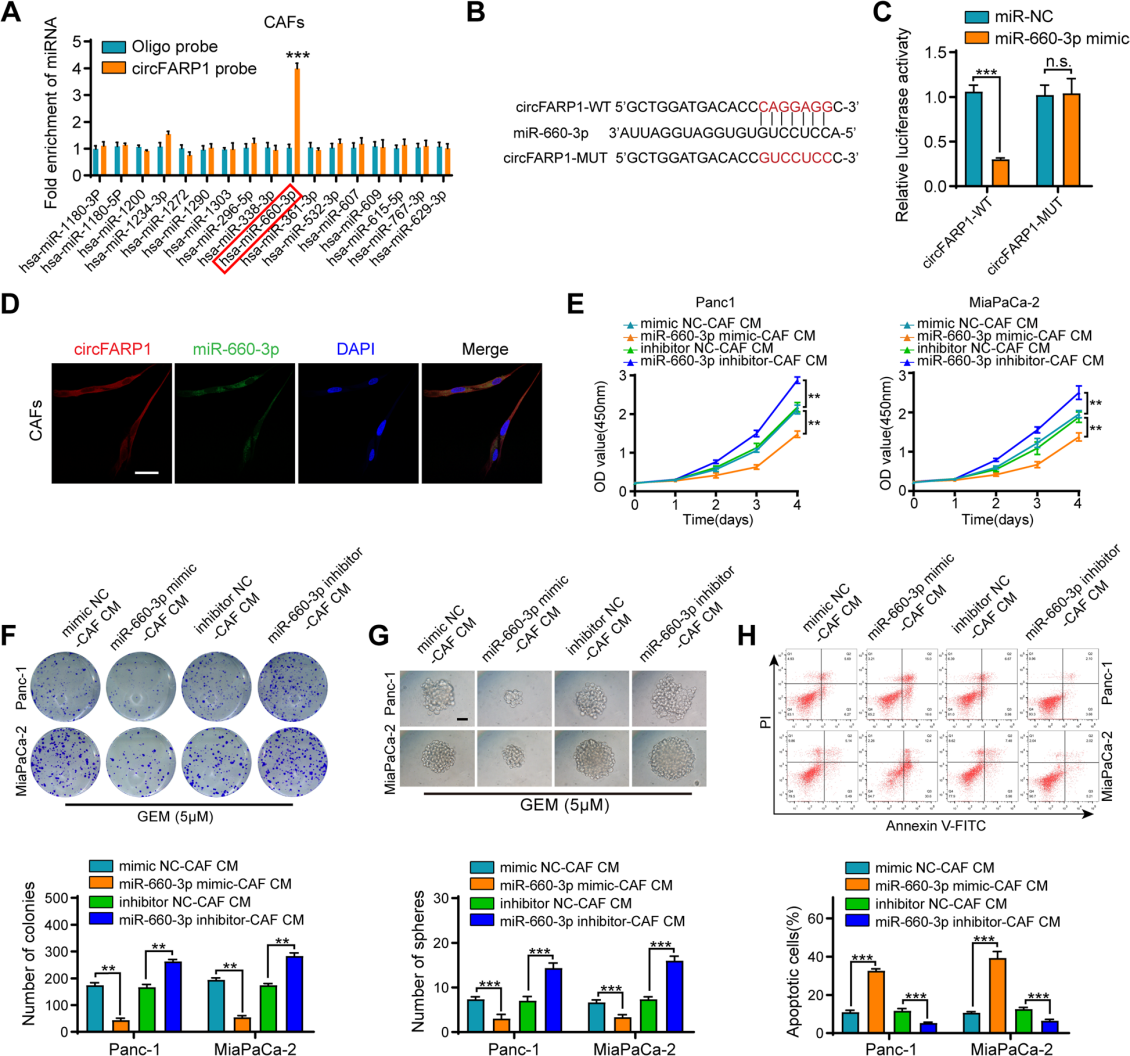
circFARP1 functions as a miR-660-3p sponge in CAFs. **A** qRT–PCR analysis of the enrichment of the indicated miRNAs in CAFs by RNA pull-down assay. **B** Schematic illustration showing the sequence alignment of circFARP1 with miR-660-3p. **C** The luciferase activities of the circFARP1 luciferase reporter plasmid (WT or MUT) following transfection with NC mimic or miR-660-3p mimics into CAFs. **D** The colocalization of circFARP1 and miR-660-3p was detected by FISH assay. Scale bar, 100μm. **E-H** Panc-1 and MiaPa-2 cells were grown in CM from CAFs transfected with miR-660-3p mimic or inhibitor for 2 weeks and then subjected to subsequent experiments. (**E**) CCK-8 assay of cell viability in Panc-1 and MiaPaCa-2 cells. (**F**) Representative images and quantification of colony formation in Panc-1 and MiaPaCa-2 cells treated with GEM (5 μM). (**G**) Representative images and quantification of sphere formation in Panc-1 and MiaPaCa-2 cells under GEM treatment (5 μM). (**H**) Flow cytometry analysis of apoptosis in Panc-1 and MiaPaCa-2 cells treated with GEM (10 μM). Data are expressed as the mean ± SD. **p<0.01 and ***p<0.001

Given that circFARP1 could enhance both the expression and secretion of LIF, we investigated another underlying mechanism contributing to enhanced LIF expression. Cytoplasm-localized circRNAs can also function as competitive endogenous RNAs (ceRNAs) and posttranscriptionally regulate target genes. Seventeen miRNAs that potentially bound to circFARP1 were predicted by CircInteractome. miRNA pulldown assays revealed that only miR-660-3p was enriched by circFARP1 in CAFs (Fig. 6A). After site-directed mutagenesis of the predicted complementary binding sites on circFARP1, miR-660-3p failed to affect the luciferase activity of circFARP1, which supported the sponge effect of circFARP1 by binding to miR-660-3p on specific sequences (Fig. 6B-C). Consistently, FISH assays confirmed the colocalization of circFARP1 and miR-660-3p in the cytoplasm of CAFs (Fig. 6D). Since an interaction between circFARP1 and miR-660-3p was determined, we further investigated whether miR-660-3p mediated GEM resistance in PDAC. We found that incubation with conditioned media from miR-660-3p-silenced CAFs enhanced the proliferation, colony formation, sphere formation, and apoptosis resistance of Panc-1 and MiaPaCa-2 cells under GEM treatment. Conversely, miR-660-3p overexpression in CAFs greatly reduced its effect in Panc-1 and MiaPaCa-2 cells (Fig. 6E-H). Consistently, the protein levels of pSTAT3, SOX2, CDA, and ABCC2 in cancer cells were increased after incubation with miR-660-3p-silenced CAFs, while miR-660-3p overexpression in CAFs reversed this effect (Fig. S6A).

**Fig. 7:**
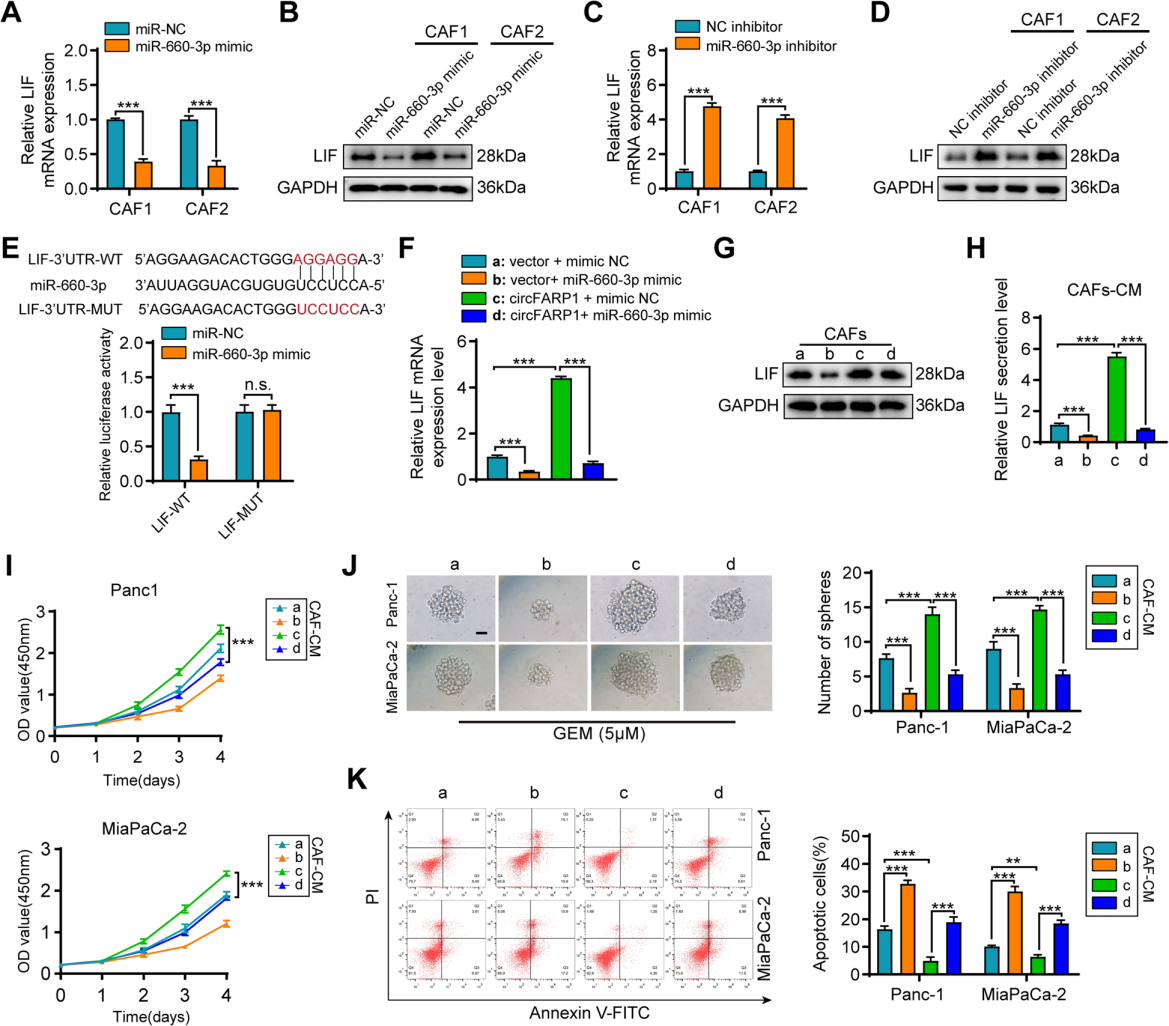
circFARP1 enhances LIF expression via miR-660-3p. **A-B** qRT–PCR and western blot analysis of LIF expression in CAF1 and CAF2 cells transfected with miR-NC or miR-660-3p mimic. **C-D** qRT–PCR and western blot analysis of LIF expression in CAF1 and CAF2 cells transfected with NC or miR-660-3p inhibitor. **E** Top panel, schematic illustrating the sequence alignment of miR-660-3p with the 3’UTR of LIF. Bottom panel, the luciferase activities of the LIF-3’ UTR luciferase reporter plasmid containing wild-type (WT) and miR-660-3p binding site mutated (Mut) and transfected with the NC mimic or miR-660-3p mimic in CAFs. **F-H** qRT–PCR, western blot and ELISA analysis of LIF expression in CAFs transfected with circFARP1 and miR-660-3p mimic alone or together. **I-K** Panc-1 and MiaPa-2 cells were grown in CM from CAFs transfected with circFARP1 and miR-660-3p mimic alone or together and then subjected to subsequent experiments. **I** The viability of Panc-1 and MiaPaCa-2 cells was detected by CCK-8. **J** Representative images and quantification of sphere formation assays on Panc-1 and MiaPaCa-2 cells under GEM treatment (5 μM). **K** Flow cytometry analysis of apoptosis in Panc-1 and MiaPaCa-2 cells treated with GEM (10 μM). Data are expressed as the mean ± SD. **p<0.01 and ***p<0.001

To validate whether circFARP1 enhanced LIF expression through miR-660-3p, we transfected a miR-660-3p mimic or miR-660-3p inhibitor into CAFs. qRT–PCR and western blotting assays showed that the mRNA and protein levels of LIF were distinctly decreased in the miR-660-3p mimic group, but upregulated in the miR-660-3p inhibitor group. (Fig. 7A-D). miRNAs suppress target gene expression by binding to the 3’ untranslated region (UTR) of their mRNAs. We found that the 3’ UTR of LIF harbored sequences complementary to part of the miR-660-3p sequence (Fig. 7E). Luciferase assays demonstrated that miR-660-3p reduced the luciferase activity of the LIF 3’ UTR luciferase construct but not the 3’ UTR luciferase construct with mutant sequences in the miR-660-3p binding site, indicating that miR-660-3p degraded LIF by directly targeting the 3’ UTR of LIF (Fig. 7E). Importantly, upregulating miR-660-3p partially abolished the effect of circFARP1 in upregulating the mRNA, protein, and secretion of LIF in CAFs (Fig. 7F-H). Consistently, upregulating miR-660-3p partially weakened the promoting effects of circFA1P1-overexpressing CAFs on proliferation, sphere formation, and apoptosis resistance under GEM treatment conditions (Fig. 7I-K). The increases in the protein levels of pSTAT3, SOX2, CDA, and ABCC2 induced by circFARP1-overexpressing CAF-derived CM were reduced by upregulating miR-660-3p (Fig. S6B). Collectively, these findings suggest that circFARP1 antagonizes the inhibitory effect of miR-660-3p on LIF expression.

### LIF is mainly secreted by CAFs in PDAC

We found that CAFs expressed higher mRNA levels of LIF than PDAC cells by using qRT–PCR assay (Fig. S7A). Surprisingly, the increased level of LIF mRNA in CAFs resulted in only slightly higher LIF protein levels than those in PDAC cells (Fig. S7B). Moreover, we detected the secretion levels of LIF and found an extremely low or even undetectable LIF concentration in media from PDAC cell lines (Fig. S7C). As CAV1 functions as a key molecule for LIF secretion, we performed western blot assays, and the results revealed that the protein levels of CAV1 were extremely low in PDAC cells (Fig. S7B). Moreover, immunostaining in PDAC samples further confirmed that few epithelial cancer cells (cytokeratin-19) expressed LIF and CAV1, whereas higher percentages of CAFs (α-SMA) were labeled with LIF and CAV1 staining (Fig. S7D-E). Collectively, these results indicated that LIF was produced by CAFs but not by tumor cells.

### Clinical implication of the circFARP1/LIF axis in PDAC

**Fig. 8:**
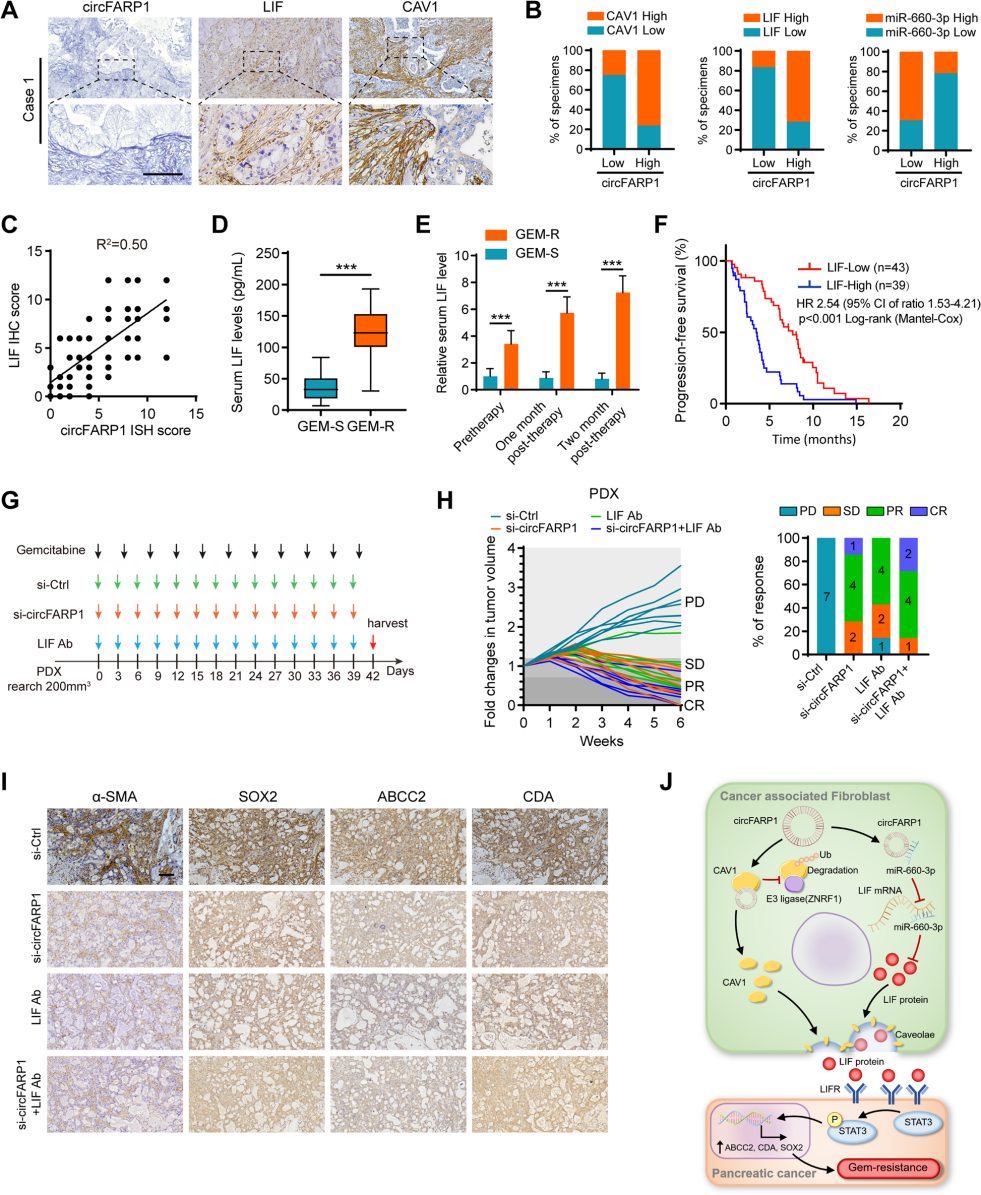
Clinical implication of the circFARP1/LIF axis in PDAC. **A** Representative images of ISH for circFARP1 and IHC for CAV1 and LIF in PDAC tissues. Scale bars, 100 μm. **B** Quantification of the percentage of specimens with low or high CAV1, miR-660-3p, and LIF in the low or high circFARP1 expression groups. **C** Correlation analysis of circFARP1 and LIF expression in PDAC tissues (*n* = 82). **D-E** ELISA analysis of the serum LIF level in GEM-S (*n* = 38) and GEM-R patients (*n* = 44). **F** Kaplan–Meier survival curves for PDAC patients who received GEM chemotherapy with high or low circFARP1 expression. **G** Timeline schematic for the treatment of mice with PDX. Colored arrows indicate the times at different treatment time points. **H** Fold changes in tumor volume and response rates in PDXs that received the indicated treatments (*n* = 7). The chemotherapy responses were evaluated using the RECIST standard. **I** Representative images of IHC for α-SMA, SOX2, ABCC2, and CDA in the PDX. Scale bars, 50 μm. **J** Schematic illustration of the mechanism by which circFARP1 enables CAFs to promote GEM chemoresistance in pancreatic cancer. Data are expressed as the mean ± SD. ***p<0.001

To investigate the clinical relevance of the circFARP1/LIF axis in PDAC, we obtained clinical samples from a cohort of 82 advanced PDAC patients who received chemotherapy with GEM. Then, circFARP1 and miR-660-3p expression was detected by FISH, and CAV1 and LIF expression was detected by IHC. The results indicated that the circFARP1-high group exhibited higher levels of LIF and CAV1 and lower levels of miR-660-3p. Conversely, the circFARP1-low group showed the opposite pattern (Fig. 8A-B, Fig. S8A-B). Furthermore, circFARP1 was positively correlated with LIF expression (Fig. 8C). Continuous monitoring of serum LIF levels in PDAC patients revealed GEM-resistant patients with higher LIF levels than GEM-sensitive patients (Fig. 8D-E). Interestingly, serum LIF levels gradually increased with GEM chemotherapy in GEM-resistant patients (Fig. 8E). Kaplan–Meier analysis showed that patients in the LIF-high group exhibited poorer PFS than those in the circFARP1-low group (Fig. 8F).

To evaluate the potential therapeutic value of the circFARP1/LIF axis in a more patient-relevant *in vivo* model of patient-derived xenografts, we created PDX models from GEM-resistant patients and performed experimental therapy with GEM and LIF antibodies and *in vivo*-optimized si-circFARP1, a circFARP1 inhibitor (Fig. 8G). Strikingly, combined treatment of the mice with GEM and si-circFARP1/anti-LIF neutralizing antibody improved the chemotherapeutic response (Fig. 8H). IHC analysis further confirmed that si-circFARP1/anti-LIF significantly reduced SOX2, CDA, and ABCC2 expression (Fig. 8I). Collectively, we demonstrated that circFARP1/LIF played an important role in mediating GEM resistance in advanced PDAC patients and highlighted the prognostic value of circFARP1 for GEM resistance (Fig. 8J).

## Discussion

Chemotherapy resistance remains a formidable challenge in pancreatic cancer. As one of the critical components of the tumor stroma, CAFs confer substantial resistance to GEM therapeutics by creating physical barriers for drug delivery and activating biochemical signaling [27]. We recently determined that CAFs modulated the GEM resistance of PDAC via the TGF-β1/SMAD2/3/ABCC1 signaling axis [28] and further revealed an epigenetic modification role of lethal giant larvae homolog 1 (LLGL1) in GEM resistance by modulating the phosphorylation of extracellular signal-regulated kinase 2 (ERK2) and specificity protein 1 (Sp1) [29]. However, little is known about the role of circRNAs in TME-mediated GEM resistance in PDAC. Herein, we characterized differentially expressed circRNAs in primary isolated fibroblasts and identified that circFARP1 was highly upregulated in CAFs and that its enrichment in tumor tissues was significantly correlated with GEM resistance in PDAC. Overexpression of circFARP1 in CAFs confers GEM resistance in pancreatic cancer cells by enhancing the expression and secretion of LIF in CAFs, thereafter activating the STAT3 signaling pathway and increasing the expression of several GEM resistance-associated factors, including ABCC2, CDA and SOX2. Mechanistically, circFARP1 modulates the secretion of LIF by directly interacting with CAV1 and inhibiting its degradation. circFARP1 also functions as a miR-660-3p sponge and increases the expression of LIF in CAFs. Furthermore, our clinical data highlighted the prognostic value of the circFARP1/CAV1/miR-660-3P/LIF axis for predicting GEM resistance in patients with pancreatic cancer. To our knowledge, this is the first report to provide insight into the biological significance of circRNA-mediated CAF-induced GEM resistance in pancreatic cancer and suggests that circFARP1 may serve as a potential therapeutic target to overcome GEM resistance in pancreatic cancer patients.

Recently, the role of circRNAs in mediating epigenetic modifications in pancreatic cancer cells has gradually been revealed [29, 30], while their underlying cancer-associated mechanism in altering the TME is far from being elucidated. By performing a pancancer analysis of 868 cancer samples, circRNA CDR1as was revealed to be a key mediator in altering the TME to promote tumor progression [31]. Kristensen et al. further verified that CDR1as was abundantly expressed in the tumor stroma but absent in cancer cells *in vivo*, which highlights the intratumor heterogeneity of circRNA expression patterns [32]. Based on our previous RNA-seq analysis of CAFs and paired NFs (GSE172096), we confirmed for the first time that circFARP1 expression in CAFs was positively correlated with GEM resistance. Two studies characterized the overall profiles of differentially expressed circRNAs correlated with GEM resistance in pancreatic cancer cells [33, 34], which was limited due to the lack of *in situ* data for potential intratumor heterogeneity. Notably, PDAC is characterized by a prominent desmoplastic reaction and comprises a fibrotic stroma accounting for up to 90% of the tumor mass [35], and the expression patterns of circRNAs in stromal cells are poorly understood. For the first time, we connected circFARP1 expression in CAFs to the GEM response of PDAC patients, suggesting the therapeutic potential of targeting circFARP1 in the TME to overcome GEM resistance in PDAC.

We explored the key role of circFARP1 in CAFs, and our gain- and loss-of-function experiments revealed that circFARP1 was critical in driving GEM resistance in pancreatic cancer cells. Gene expression profile analysis and further experiments showed that circFARP1 conferred GEM resistance by enhancing LIF expression and secretion in CAFs, thereafter increasing the expression of ABCC2, CDA and SOX2 by activating the STAT3 signaling pathway in pancreatic cancer cells. Previous reports have shown the role of exosomal circRNAs from CAFs in mediating cancer progression [36, 37]. Without enrichment in exosomes (data not shown). circFARP1 was mainly localized in the cytoplasm and mediated the expression and secretion of the key cytokine LIF in CAFs in our study. As a pluripotent cytokine, LIF regulates cell differentiation, proliferation and survival during embryonic development and disease progression [38]. Recently, LIF has been corroborated as a predominant paracrine factor from activated pancreatic stellate cells and was found to be involved in PDAC pathogenesis [39–41]. Regarding the specific source of LIF, our results found that the expression of LIF in pancreatic cancer cell lines was significantly lower than that of CAFs. Impressively, we could hardly detect the secretion of LIF in the supernatant of pancreatic cancer cells. Consistent with our results, Shi et al revealed that LIF was produced by human pancreatic stellate cells in large amounts, but not by MiaPaCa-2 [39]. As a member of the IL-6 superfamily of cytokines, LIF forms a heterodimer with a specific LIFR and gp130 to activate the STAT3 pathway [42]. We found that the circFARP1/LIF axis was essential for the continuous activation of pSTAT3. Consistently, Yu et al. confirmed that either LIFR knockdown in pancreatic cells or LIF ligand neutralization from the CM of pancreatic stellate cells effectively repressed STAT3 activation [42]. We further determined that the circFARP1/LIF axis remarkably increased the expression of GEM resistance-related genes, such as ABCC2, CDA, and SOX2, and induced GEM resistance in pancreatic cancer cells. Intriguingly, Yu et al. demonstrated that LIFR-deficient pancreatic cancer cells are more sensitive to GEM and that the intrinsic GEM resistance of tumor-initiating cells relies on LIFR signaling [39]. LIF levels have been linked to PDAC differentiation status, intratumoral nerve density and overall survival [39, 40]. We are the first to report the clinical implication of the circFARP1//LIF axis in the GEM response in PDAC. Encouragingly, the first-in-human clinical trial that combined the anti-LIFR antibody EC359 and GEM to target pancreatic tumor stroma and cancer cells was supported by the National Institute of Health [43]. As such, the novel circFARP1/LIF axis may provide additional therapeutic targets and biomarkers in PDAC that can be translated to the clinic and warrants further clinical evaluation.

To gain mechanistic insights into circFARP1, we studied the circFARP1-binding proteome and identified CAV1 as the protein partner involved in GEM resistance. Without altering CAV1 mRNA expression levels, we found that circFARP1 directly bound to CAV1 and blocked the interaction between CAV1 and E3 ligases to suppress CAV1 ubiquitination. As a key factor for cytokine trafficking, CAV1 enhanced the secretion of LIF in CAFs. In addition, we found that circFARP1 functions as a miR-660-3p sponge and antagonizes the inhibitory effect of miR-660-3p on LIF expression. The functional role of LIF in TME-mediated cancer progression has been well characterized, but little is known about its upstream modulators in CAFs. A recent study showed that LIF was the key mediator for maintaining the inflammatory CAF phenotype in pancreatic cancer, which was upregulated by tumor-secreted IL-1-induced NF-κB signaling activation in CAFs [41]. In addition, Zhang et al showed that miR-637 directly bound to the LIF 3’ UTR and suppressed LIF expression, thereby inhibiting tumorigenesis in hepatocellular carcinoma by blocking Stat3 phosphorylation [44]. Similarly, Zheng et al. demonstrated that upregulated circSEPT9 in triple-negative breast cancer could enhance the mRNA level of LIF by sponging miR-637, which contributed to the activation of LIF/Stat3 signaling [45]. Consistently, our results revealed that ceRNA network disruption was also the key process for LIF upregulation in CAFs, and our finding first exposed the circFARP1/miR-660-3p sponge as a crucial upstream mediator of LIF. Remarkably, the versatile role of circRNAs in posttranscriptional regulation has been gradually revealed in recent years. A previous study demonstrated that circ-CCAC1 elevated YY1 expression to promote cholangiocarcinoma (CCA) by sponging miR-514a-5p in CCA cells while elevating SH3GL2 expression to enhance cell permeability by directly sequestering EZH2 in the cytoplasm of human umbilical vein endothelial cells [46]. Depending on the different subcellular locations, circACTN4 could interact with YBX1 to coactivate the transcription of FZD7 in the nucleus and sponge miR-424-5p to upregulate the mRNA level of YAP1 in the cytoplasm, thereby facilitating the development and progression of intrahepatic cholangiocarcinoma [47]. Interestingly, we revealed the dual mechanism of circRNA in the cytoplasm; this was the study report to reveal that circFARP1 accurately and cooperatively regulates the expression and secretion of LIF, implying that circRNAs collaborate in the TME to drive tumor chemoresistance.

A previous study showed that although LIF expression was upregulated in both cancer cells and stromal cells of PDAC, it was only secreted from stromal cells [40], suggesting the specific mechanism of LIF secretion in the TME. Our study has extended the knowledge concerning topics from the expression pattern to the secretion mode of LIF in CAFs of PDAC. We demonstrated that circFARP1-induced CAV1 is an essential membrane structural factor that mediates the secretion of LIF. As the essential structural component of caveolae, CAV1 is a key scaffolding protein in cellular trafficking [48]. Previous studies have shown that the upregulation of CAV1 is associated with a poor prognosis, a more aggressive subtype and GEM resistance in PDAC [49–51]. Interestingly, Adam et al. recently observed that the elevation of CAV1 in PDAC cells after GEM treatment subsequently increased albumin uptake, leading to maximal treatment efficacy with a novel schedule of GEM followed by nanoparticle albumin-bound paclitaxel treatment at 48 hours, and proposed that low Cav-1 expressing tumors would stand to benefit most from this schedule [52]. The relationship of CAV1 expression in CAFs and clinical prognosis and biological significance may vary according to the kind of cancer. Previous studies showed the associations of high expression of CAV1 in CAFs with cancer progression and a poor prognosis in patients with pancreatic cancer, kidney carcinoma, colon carcinoma, and melanoma [53, 54]. In contrast, the loss of stromal CAV1 induces the myofibroblast phenotype via TGFβ signaling and contributes to poor outcomes in lung and breast cancers [43, 55]. Our study determined a novel role of CAV1 in mediating the secretion of LIF by CAFs and promoting GEM resistance in PDAC. As heterogeneity in CAF fate and function has also attracted great attention, researchers have discovered two CAF phenotypes, classified as the myofibroblastic CAF (myCAF) phenotype and the inflammatory CAF (iCAF) phenotype, based on the expression levels of α-SMA [56]. The key mediators of LIF for the iCAF phenotype and TGFβ for the myCAF phenotype in pancreatic cancer are modulated by CAV1, which would explain the contradictory role of CAV1 in the TME, and the detailed mechanisms require further analysis.

## Conclusions

Altogether, our work demonstrated that circFARP1 was highly expressed in CAFs and that its enrichment in tumor tissues was positively correlated with GEM resistance and poor survival in a cohort of advanced PDAC patients. circFARP1 functions as a ceRNA by sponging miR-660-3p to increase the expression of LIF and synergistically enhances LIF secretion by directly binding with CAV1 to inhibit the degradation of CAV1 by blocking the interaction of CAV1 and the E3 ubiquitin ligase ZNRF1, thereby activating the STAT3 signaling pathway in pancreatic cancer cells to induce GEM resistance in PDAC. This is the first report to reveal the biological processes of circRNA-mediated LIF paracrine signaling by CAFs to establish a distinct fibroblast niche mediating GEM resistance in PDAC, suggesting the urgent need for the development of rational strategies that target the circFARP1/CAV1/miR-660-3p/LIF axis in the TME to overcome GEM resistance in pancreatic cancer patients.

## Supplementary Information

**Additional file 1: Figure S1.** Screening circFARP1 is associated with GEM chemoresistance. Related to Fig. 1. (A-B) qRT–PCR analysis of circCUL2 and circARMC9 expression in NFs, CAFs, primary cancer cells, macrophages, and PDAC cell lines. (C-D) Quantification of circCUL2 and circARMC9 expression by using qRT–PCR in GEM-S (n = 38) and GEM-R (n = 44) PDAC tissues. The left panel shows the plot of circCUL2 and circARMC9 expression in each tissue. Right panel shows the expression as violin plots. Data are expressed as the mean ± SD, n.s., no significant.

**Additional file 2: Figure S2.** Specificity of circCUL2 shRNA and overexpression vector. Related to Fig. 2. (A-B) qRT–PCR analysis of circCUL2 and CUL2 expression following transfection of circCUL2 shRNA and overexpression vector. Data are expressed as the mean ± SD. ***p<0.001.

**Additionsl file 3: Figure S3.** circFARP1 enhanced the distinct migration properties of CAFs. CAFs were stably transfected with empty vector, circFARP1, lenti-NC-shRNA, or lenti-circFARP1-shRNA and then subjected to the indicated experiments. (A-B) Morphology of the indicated CAFs under a light microscope. Cells grew mainly in clusters of spindle or polygonal shape. Scale bars, 100 μm. (C-D) Cell proliferation of indicated CAFs. (E-F) Representative images and quantification of EdU-incorporating CAFs. Scale bars, 100 μm. (G-H) Scratch wound healing assay of the indicated CAFs. Scale bars, 100 μm. (I-J) Representative images and quantification of Transwell migration assays for the indicated CAFs. Scale bars, 100 μm. (K-L) Representative photographs of collagen gel contraction by the indicated CAFs. Data are expressed as the mean ± SD. n.s., no significant. ***p<0.001.

**Additional file 4: Figure S4.** circFARP1 enhances the expression and secretion of LIF in CAFs. Related to Fig. 3. (A) qRT–PCR analysis of candidate downstream targets of circFARP1. (B-C). The mRNA level (B) and secretion level (C) of LIF in CAFs transfected with sh-NC or sh-circFARP1. (D) Tumor weight were shown. (E) Quantification of Ki67-positive cells in subcutaneous tumors. (F) IHC staining of subcutaneous tumors with antibodies specific for Ki67. Scale bars, 100μm. Data are expressed as the mean ± SD. ***p<0.001.

**Additional file 5: Figure S5.** The effect of CAV1 in activating STAT3 pathway. Related to Fig. 5. (A) Panc-1 and MiaPaCa-2 cells were treated with CM from CAFs transfected with lenti-NC-shRNA or lenti-CAV1-shRNA for 2 weeks. Western blot analysis of pstat3/stat3, ABCC2, CDA, and SOX2 protein expression in the indicated Panc-1 and MiaPaCa-2 cells. (B). Panc-1 and MiaPa-2 cells were grown in CM from CAFs transfected with lenti-circFARP1 and lenti-CAV1-shRNA alone or together for 2 weeks. Western blot analysis of pstat3/stat3, ABCC2, CDA, and SOX2 protein expression in the indicated Panc-1 and MiaPaCa-2 cells.

**Additional file 6: Figure S6.** The effect of miR-660-3p in activating STAT3 pathway. Related to Fig. 7. (A) Panc-1 and MiaPa-2 cells were grown in CM from CAFs transfected with miR-660-3p mimic or inhibitor for 2 weeks. Western blot analysis of pstat3/stat3, ABCC2, CDA, and SOX2 protein expression in Panc-1 and MiaPaCa-2 cells. (B) Panc-1 and MiaPa-2 cells were grown in CM from CAFs transfected with circFARP1 and miR-660-3p mimic alone or together. Western blot analysis of pstat3/stat3, ABCC2, CDA, and SOX2 protein expression in the indicated Panc-1 and MiaPaCa-2 cells.

**Additional file 7: Figure S7.** LIF is mainly secreted by CAFs. (A) LIF mRNA expression levels in CAFs and Panc-1 and MiaPaCa-2 cells. (B) western blot analysis of LIF and CAV1 in CAFs and Panc-1 and MiaPaCa-2 cells. (C) ELISA of LIF in CAFs and Panc-1 and MiaPaCa-2 cells. (D) Representative images of LIF, CK19, and α-SMA immunostaining in human PDAC specimens. Scale bar, 100μm. (E) Representative images of CAV1, CK19, and α-SMA immunostaining in human PDAC specimens. Scale bar, 100 μm.

**Additional file 8: Figure S8.** Clinical implication of the circFARP1//LIF axis in PDAC. (A-B) Representative images of ISH for circFARP1, FISH for miR-660-3p, and IHC for CAV1 and LIF in PDAC tissues. Scale bars, 100 μm.

**Additional file 9: Table S2.** Oligonucleotide sequences for this study.

**Additional file 10: Table S1.** Primers used in PCR.

**Additional file 11.**

## Data Availability

The datasets used in current study are available from the corresponding author on reasonable request.

## Abbreviations

CAFs: Cancer-associated fibroblasts
GEM: Gemcitabine
PDAC: Pancreatic ductal adenocarcinoma
circRNAs: Circular RNAs
TME: Tumor microenvironment
ECM: Extracellular matrix
LIF: Leukemia inhibitory factor
NFs: Normal fibroblasts
PFS: Progression-free survival
FISH: Fluorescence *in situ* hybridization
CAF-CM: CAF-derived conditioned medium
CCK-8: Cell Counting Kit-8
IC50: Inhibitory concentration
KEGG: Kyoto Encyclopedia of Genes and Genomes
IF: Immunofluorescence
CHX: Cyclohexamide
ZNRF1: Zinc and ring finger 1
ceRNAs: Competitive endogenous RNAs
UTR: Untranslated region
