# Supplementary figures and images for: circFARP1 enables cancer-associated fibroblasts to promote gemcitabine resistance in pancreatic cancer via the LIF/STAT3 axis
Source: Mol Cancer. 2022 Jan 19;21:24. doi: 10.1186/s12943-022-01501-3 (PMC8767726; doi:10.1186/s12943-022-01501-3)

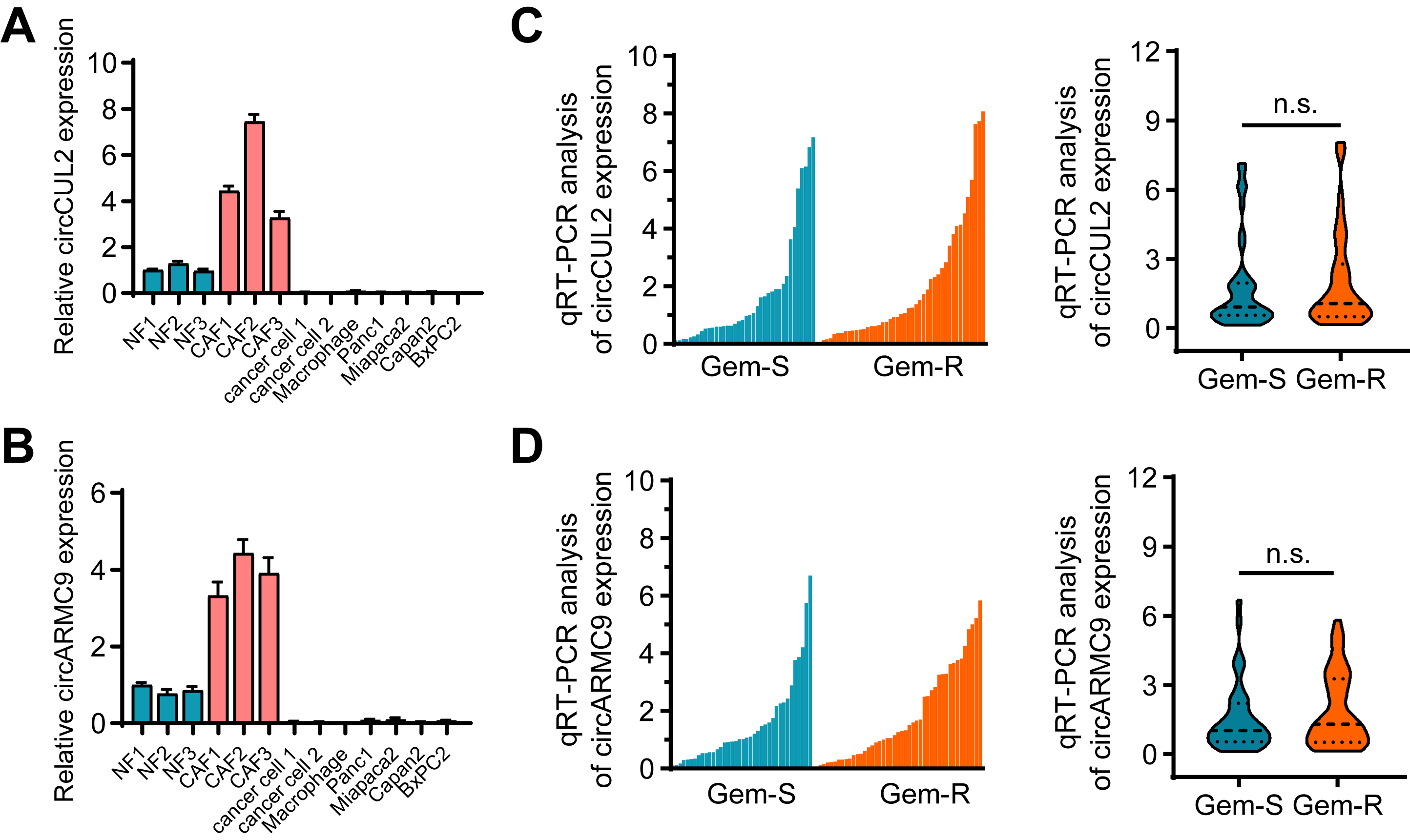

Supplement: Supplementary file 1 — Additional file 1: Figure S1. Screening circFARP1 is associated with GEM chemoresistance. Related to Fig. 1. (A-B) qRT–PCR analysis of circCUL2 and circARMC9 expression in NFs, CAFs, primary cancer cells, macrophages, and PDAC cell lines. (C-D) Quantification of circCUL2 and circARMC9 expression by using qRT–PCR in GEM-S (n = 38) and GEM-R (n = 44) PDAC tissues. The left panel shows the plot of circCUL2 and circARMC9 expression in each tissue. Right panel shows the expression as violin plots. Data are expressed as the mean ± SD, n.s., no significant. [file 12943_2022_1501_MOESM1_ESM.tif]

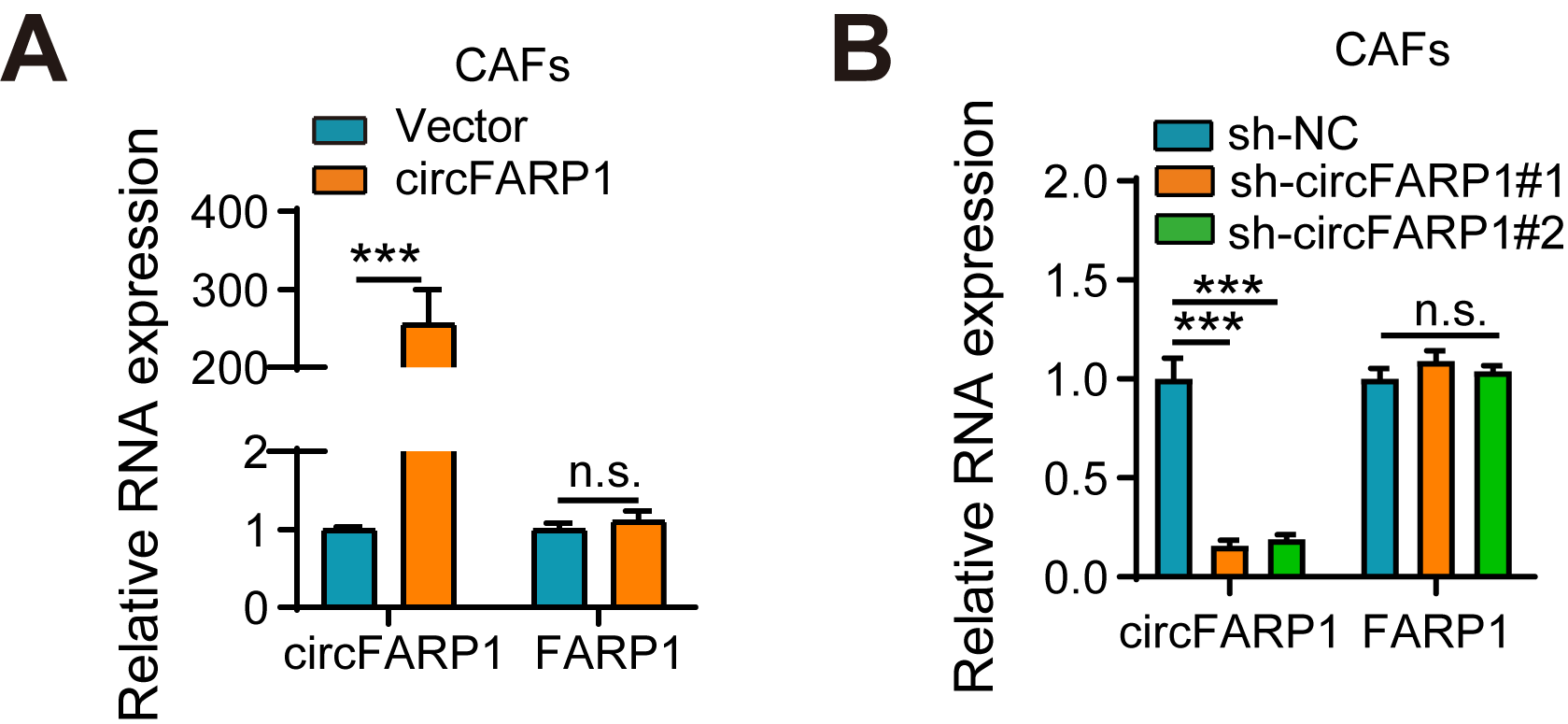

Supplement: Supplementary file 2 — Additional file 2: Figure S2. Specificity of circCUL2 shRNA and overexpression vector. Related to Fig. 2. (A-B) qRT–PCR analysis of circCUL2 and CUL2 expression following transfection of circCUL2 shRNA and overexpression vector. Data are expressed as the mean ± SD. ***p<0.001. [file 12943_2022_1501_MOESM2_ESM.tif]

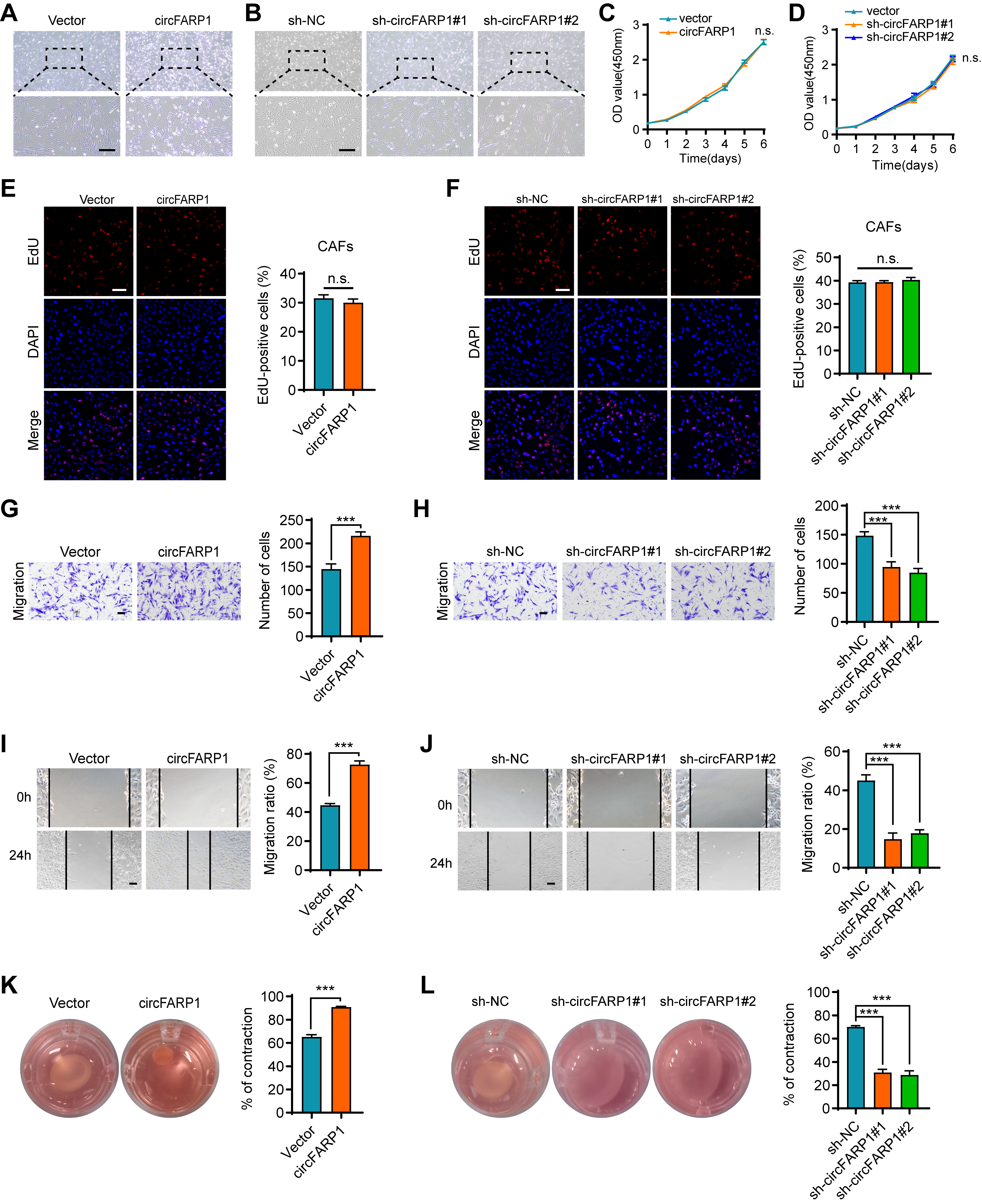

Supplement: Supplementary file 3 — Additionsl file 3: Figure S3. circFARP1 enhanced the distinct migration properties of CAFs. CAFs were stably transfected with empty vector, circFARP1, lenti-NC-shRNA, or lenti-circFARP1-shRNA and then subjected to the indicated experiments. (A-B) Morphology of the indicated CAFs under a light microscope. Cells grew mainly in clusters of spindle or polygonal shape. Scale bars, 100 μm. (C-D) Cell proliferation of indicated CAFs. (E-F) Representative images and quantification of EdU-incorporating CAFs. Scale bars, 100 μm. (G-H) Scratch wound healing assay of the indicated CAFs. Scale bars, 100 μm. (I-J) Representative images and quantification of Transwell migration assays for the indicated CAFs. Scale bars, 100 μm. (K-L) Representative photographs of collagen gel contraction by the indicated CAFs. Data are expressed as the mean ± SD. n.s., no significant. ***p<0.001. [file 12943_2022_1501_MOESM3_ESM.tif]

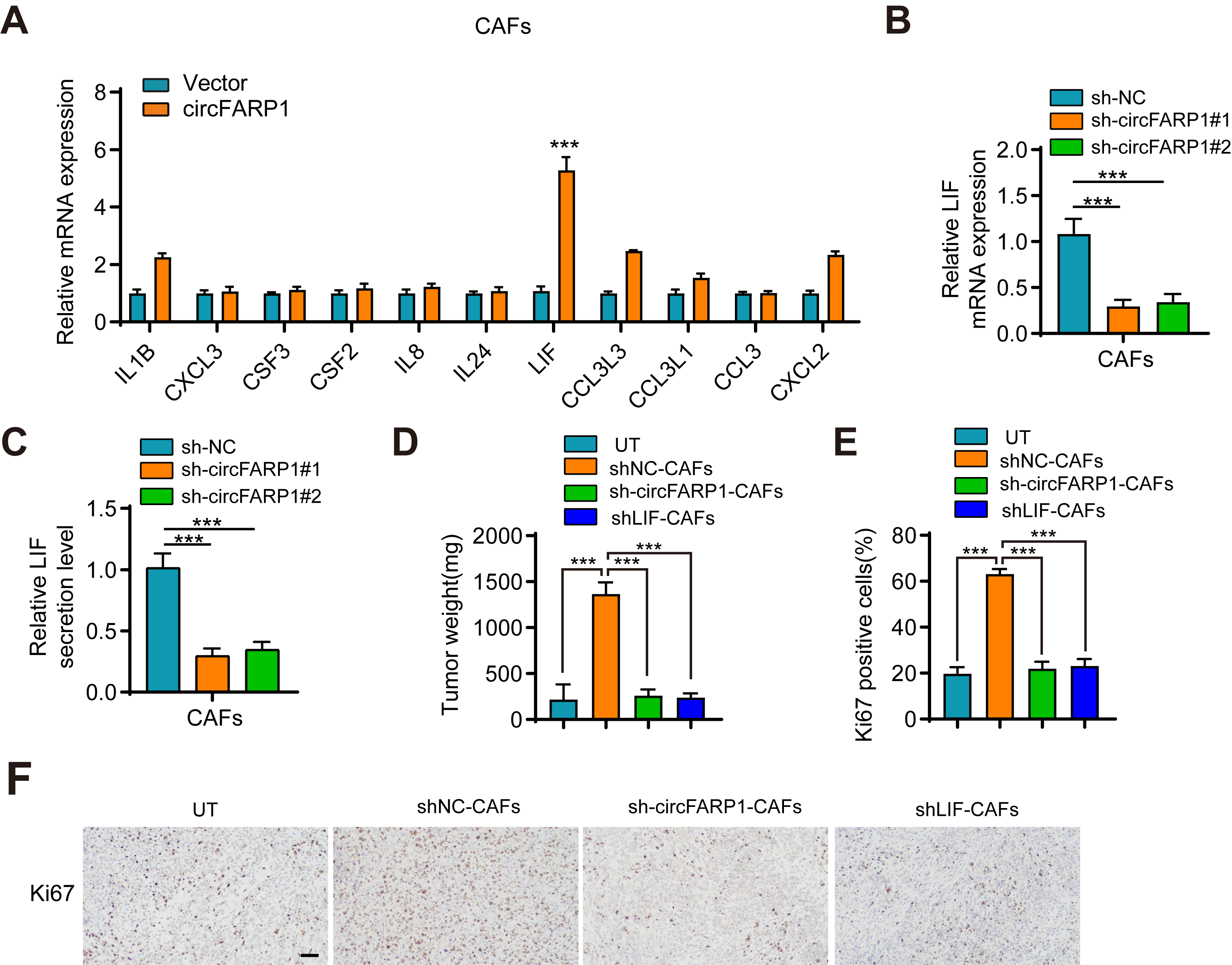

Supplement: Supplementary file 4 — Additional file 4: Figure S4. circFARP1 enhances the expression and secretion of LIF in CAFs. Related to Fig. 3. (A) qRT–PCR analysis of candidate downstream targets of circFARP1. (B-C). The mRNA level (B) and secretion level (C) of LIF in CAFs transfected with sh-NC or sh-circFARP1. (D) Tumor weight were shown. (E) Quantification of Ki67-positive cells in subcutaneous tumors. (F) IHC staining of subcutaneous tumors with antibodies specific for Ki67. Scale bars, 100μm. Data are expressed as the mean ± SD. ***p<0.001. [file 12943_2022_1501_MOESM4_ESM.tif]

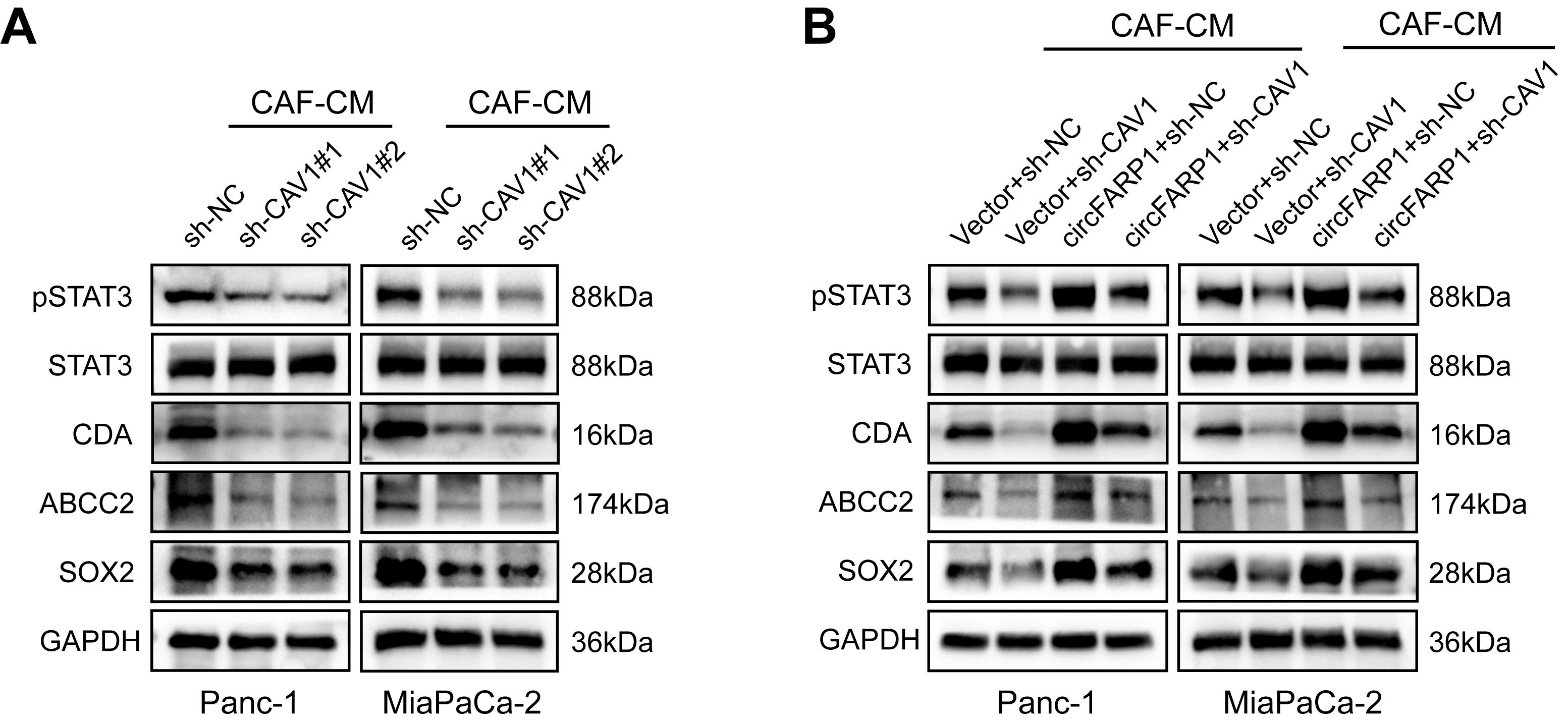

Supplement: Supplementary file 5 — Additional file 5: Figure S5. The effect of CAV1 in activating STAT3 pathway. Related to Fig. 5. (A) Panc-1 and MiaPaCa-2 cells were treated with CM from CAFs transfected with lenti-NC-shRNA or lenti-CAV1-shRNA for 2 weeks. Western blot analysis of pstat3/stat3, ABCC2, CDA, and SOX2 protein expression in the indicated Panc-1 and MiaPaCa-2 cells. (B). Panc-1 and MiaPa-2 cells were grown in CM from CAFs transfected with lenti-circFARP1 and lenti-CAV1-shRNA alone or together for 2 weeks. Western blot analysis of pstat3/stat3, ABCC2, CDA, and SOX2 protein expression in the indicated Panc-1 and MiaPaCa-2 cells. [file 12943_2022_1501_MOESM5_ESM.tif]

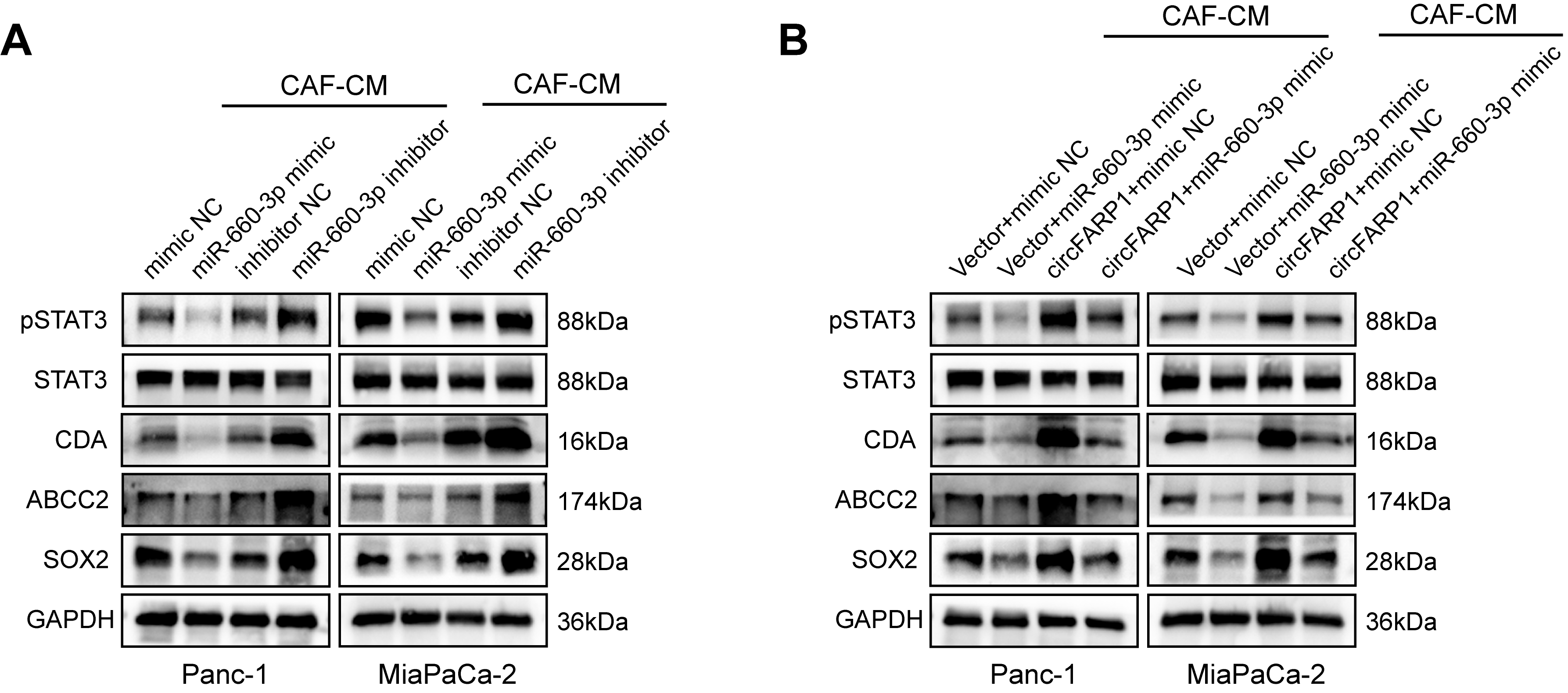

Supplement: Supplementary file 6 — Additional file 6: Figure S6. The effect of miR-660-3p in activating STAT3 pathway. Related to Fig. 7. (A) Panc-1 and MiaPa-2 cells were grown in CM from CAFs transfected with miR-660-3p mimic or inhibitor for 2 weeks. Western blot analysis of pstat3/stat3, ABCC2, CDA, and SOX2 protein expression in Panc-1 and MiaPaCa-2 cells. (B) Panc-1 and MiaPa-2 cells were grown in CM from CAFs transfected with circFARP1 and miR-660-3p mimic alone or together. Western blot analysis of pstat3/stat3, ABCC2, CDA, and SOX2 protein expression in the indicated Panc-1 and MiaPaCa-2 cells. [file 12943_2022_1501_MOESM6_ESM.tif]

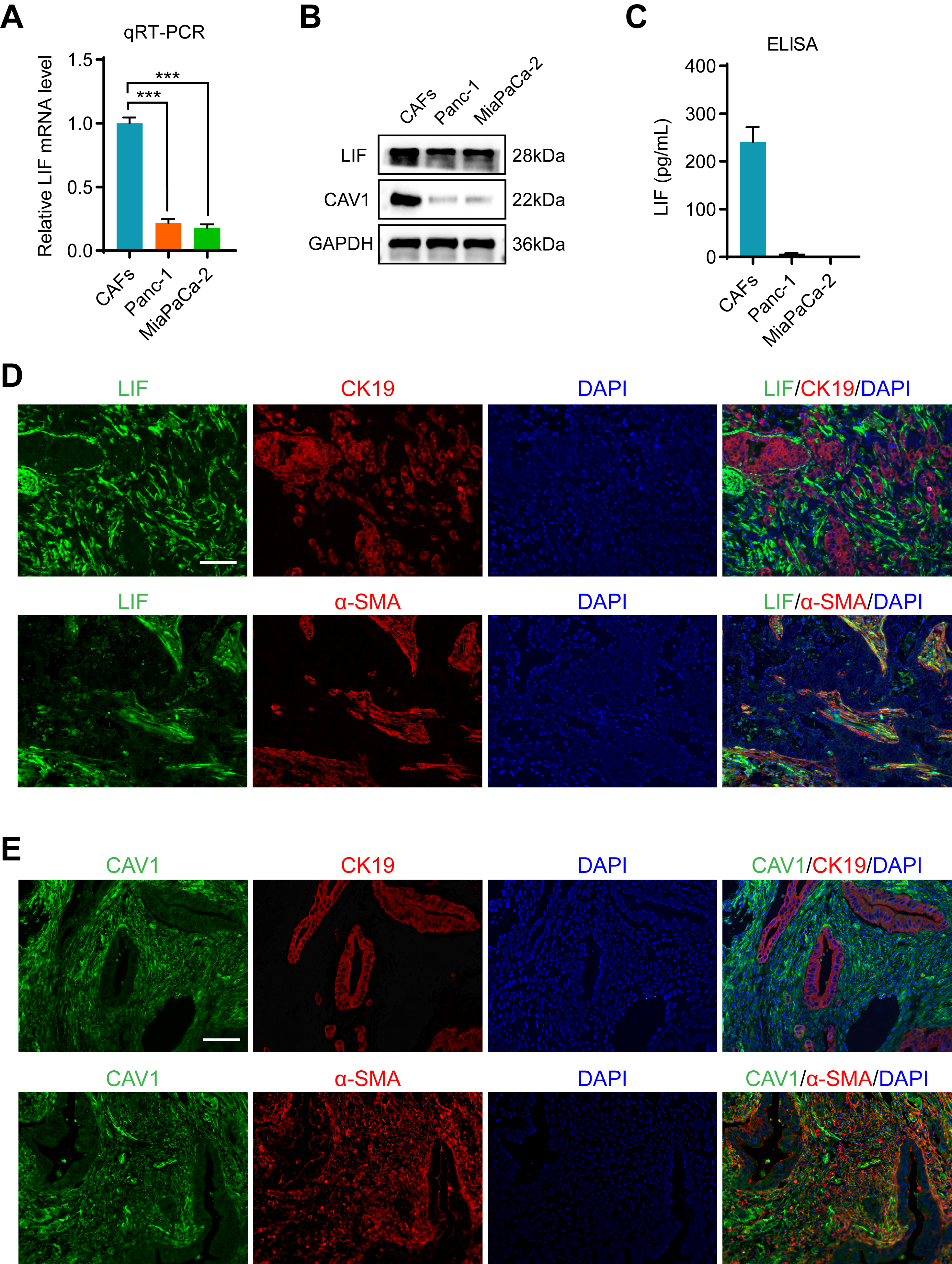

Supplement: Supplementary file 7 — Additional file 7: Figure S7. LIF is mainly secreted by CAFs. (A) LIF mRNA expression levels in CAFs and Panc-1 and MiaPaCa-2 cells. (B) western blot analysis of LIF and CAV1 in CAFs and Panc-1 and MiaPaCa-2 cells. (C) ELISA of LIF in CAFs and Panc-1 and MiaPaCa-2 cells. (D) Representative images of LIF, CK19, and α-SMA immunostaining in human PDAC specimens. Scale bar, 100μm. (E) Representative images of CAV1, CK19, and α-SMA immunostaining in human PDAC specimens. Scale bar, 100 μm. [file 12943_2022_1501_MOESM7_ESM.tif]

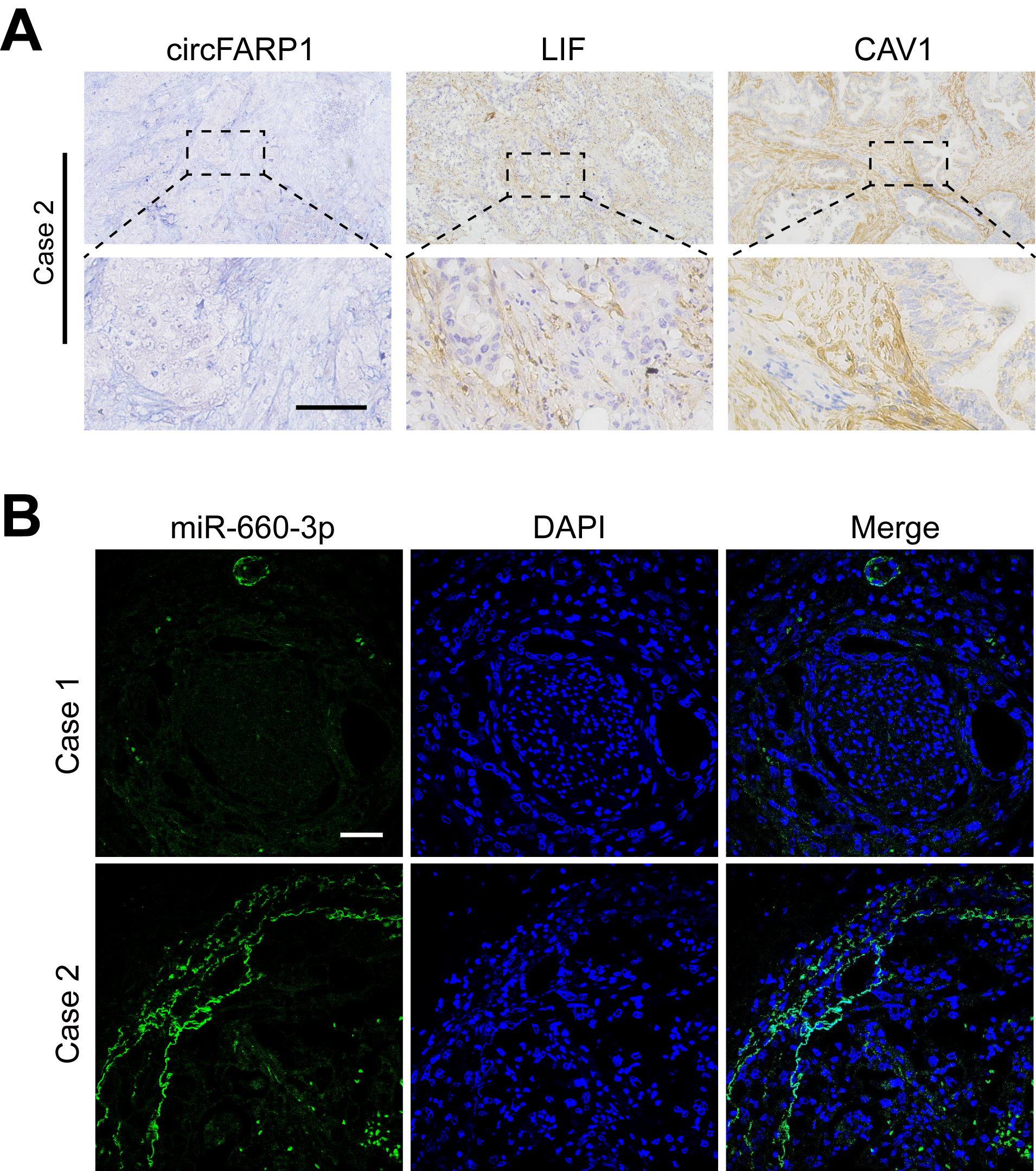

Supplement: Supplementary file 8 — Additional file 8: Figure S8. Clinical implication of the circFARP1//LIF axis in PDAC. (A-B) Representative images of ISH for circFARP1, FISH for miR-660-3p, and IHC for CAV1 and LIF in PDAC tissues. Scale bars, 100 μm. [file 12943_2022_1501_MOESM8_ESM.tif]

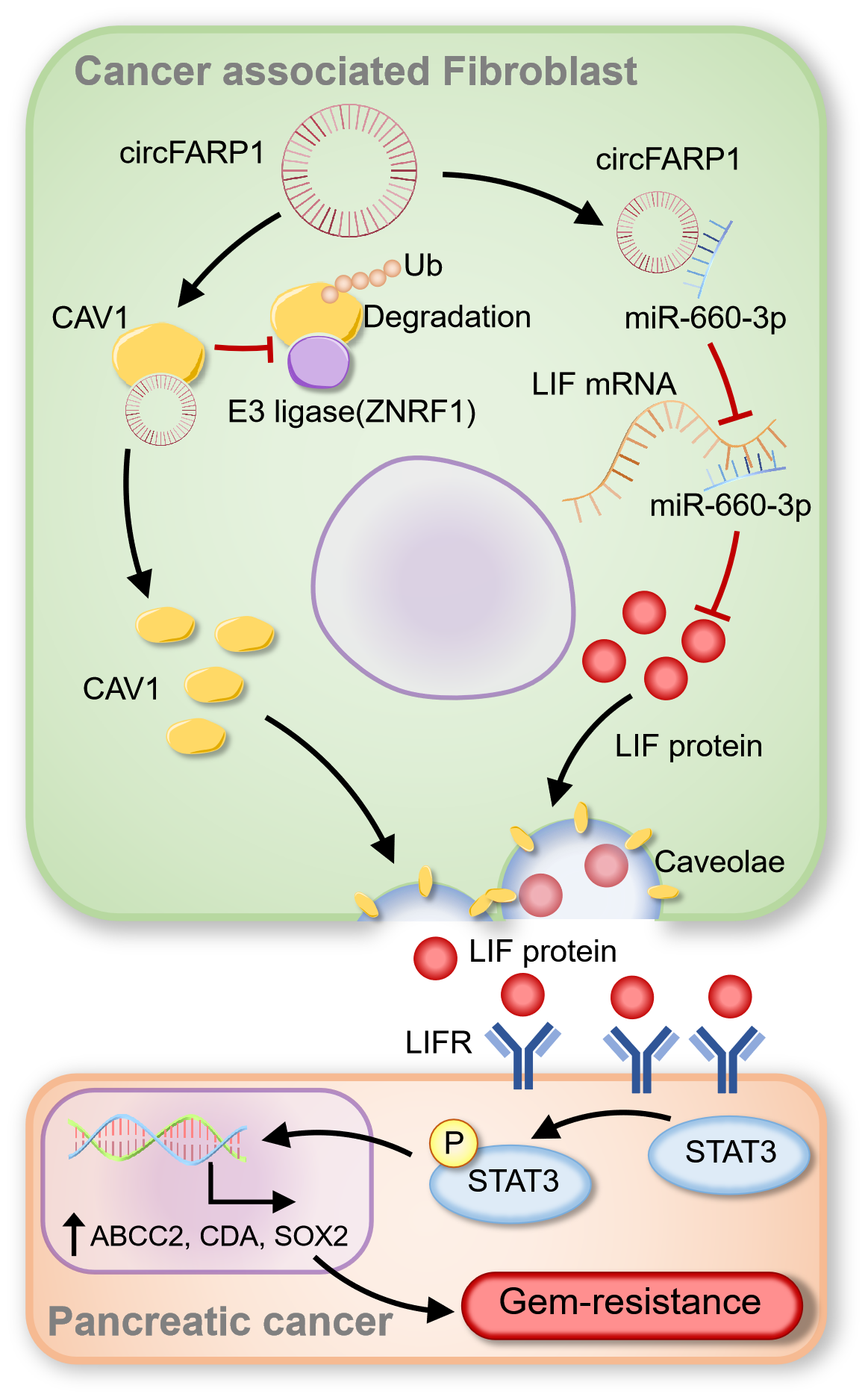

Supplement: Supplementary file 11 — Additional file 11. [file 12943_2022_1501_MOESM11_ESM.tif]
