# Supplementary material for: circFARP1 enables cancer-associated fibroblasts to promote gemcitabine resistance in pancreatic cancer via the LIF/STAT3 axis
Source: Mol Cancer. 2022 Jan 19;21:24. doi: 10.1186/s12943-022-01501-3 (PMC8767726; doi:10.1186/s12943-022-01501-3)
Supplement: Supplementary file 9 — Additional file 9: Table S2. Oligonucleotide sequences for this study. [file 12943_2022_1501_MOESM9_ESM.docx]

**Table S2. Oligonucleotide sequences for this study.**

| **Name** | **Sequence** |
| --- | --- |
| **siRNA/shRNA** | |
| sh-NC | 5’TTCTCCGAACGTGTCACGT3’ |
| sh-circFARP1 #1 | 5’GGCATTTGAAGTTCCAATATT3’ |
| sh-circFARP1 #2 | 5’GAAGTTCCAATATTCTCTAAG3’ |
| sh-LIF | 5’CAACAACCTGGACAAGCTATG3’ |
| sh-CAV1#1 | 5’CCAGAAGGGACACACAGTT3’ |
| sh-CAV1#2 | 5’GCCGTGTCTATTCCATCTA3’ |
| miR-660-3p mimic | 5’ACCUCCUGUGUGCAUGGAUUA3’ |
| miR-660-3p inhibitor | 5’UAAUCCAUGCACACAGGAGGU3’ |
| In vivo siRNA | 5'CAUUUGAAGUUCCAAUAUUdTdT3’ |
| **Probe for RNA pull down, FISH/ISH** | |
| CircFARP1 probe | AAGCGGCUUAGAGAAUAUUGGAACUUCAAAUGCCUCCU |
| miR-660-3p probe | UAAUCCAUGCACACAGGAGGU |
