# Supplementary material for: circFARP1 enables cancer-associated fibroblasts to promote gemcitabine resistance in pancreatic cancer via the LIF/STAT3 axis
Source: Mol Cancer. 2022 Jan 19;21:24. doi: 10.1186/s12943-022-01501-3 (PMC8767726; doi:10.1186/s12943-022-01501-3)
Supplement: Supplementary file 10 — Additional file 10: Table S1. Primers used in PCR. [file 12943_2022_1501_MOESM10_ESM.docx]

**Table S1. Primers used in PCR.**

| **Primer Name** | **Sense (+)**  **Antisense (-)** | **Sequence** |
| --- | --- | --- |
| CircFARP1 | **+** | 5’-CCAACACCTTCAGGAAAACTCG-3’ |
|  | **-** | 5’-GCGGCTTAGAGAATATTGGAACT-3’ |
| FARP1 | **+** | 5’- AGTTTGCAACCACCTCAACC -3’ |
|  | **-** | 5’- AACAACGTGCTTTGGCCTTC -3’ |
| miR-660-3p-specific primer | **+** | 5’-CGACCTCCTGTGTGCATGGATTA-3’ |
| LIF | **+** | 5’-CCCTGGTCCCTACTCAACAA-3’ |
|  | **-** | 5’-CTGGACCCTGACACCCTAAA-3’ |
| CAV1 | + | 5’- AGAAAGAAGATGGGGGAGGA -3’ |
|  | - | 5’- CCCAAAGGCAGAATCACAAT -3’ |
| CSF3 |  | 5’-ACGAGGGTCAGGACTGTGAC-3’ |
|  |  | 5’-GTGACAGTGGAGGGGACACT-3’ |
| IL1B |  | 5’-GGGCCTCAAGGAAAAGAATC-3’ |
|  |  | 5’-TTCTGCTTGAGAGGTGCTGA-3’ |
| CXCL3 |  | 5’-GCAGGGAATTCACCTCAAGA-3’ |
|  |  | 5’-GGTGCTCCCCTTGTTCAGTA-3’ |
| CSF2 |  | 5’-TTCTGCTTGTCATCCCCTTT-3’ |
|  |  | 5’-TGCCTGTATCAGGGTCAGTG-3’ |
| IL8 |  | 5’-TAGCAAAATTGAGGCCAAGG-3’ |
|  |  | 5’-GGACTTGTGGATCCTGGCTA-3’ |
| IL24 |  | 5’-GAACCTTCCACCCACAGCTA-3’ |
|  |  | 5’-CCAAGCAGCCTCAATTCTTC-3’ |
| CCL3L3 |  | 5’-CTCTCTGCAACCAGGTCCTC-3’ |
|  |  | 5’-TTTCTGGACCCACTCCTCAC-3’ |
| CCL3L1 |  | 5’-GTCCTTTCTTGGCTCTGCTG-3’ |
|  |  | 5’-TGGCTGCTCGTCTCAAAGTA-3’ |
| CCL3 |  | 5’-AGTGAGGAGTGGGTCCAGAA-3’ |
|  |  | 5’-TTGGCAACAACCAGTCCATA-3’ |
| CXCL2 |  | 5’-CACACTCAAGAATGGGCAGA-3’ |
|  |  | 5’-AAACACATTAGGCGCAATCC-3’ |
| GAPDH  (convergent) | + | 5’-GTCATCCCTGAGCTGAACGG-3’ |
|  | **-** | 5’-GTCAAAGGTGGAGGAGTGGG-3’ |
| GAPDH  (divergent) | **+** | 5’-CACCACACTGAATCTCCCCT-3’ |
|  | **-** | 5’-ATTTCCTTCCCGGTTGCAAC-3’ |
| CircFARP1  (convergent) | **+** | 5’-AAGCCGCTTTCATCATGGGA-3’ |
|  | **-** | 5’-TCAAATGCCTCCTGGGTGTC-3’ |
